# COVID-19 epidemic modelling for policy decision support in Victoria, Australia 2020–2021

**DOI:** 10.1186/s12889-023-15936-w

**Published:** 2023-05-27

**Authors:** Nick Scott, Romesh G Abeysuriya, Dominic Delport, Rachel Sacks-Davis, Jonathan Nolan, Daniel West, Brett Sutton, Euan M Wallace, Margaret Hellard

**Affiliations:** 1 Disease Elimination Program, Burnet Institute, 85 Commercial Rd, Melbourne, Victoria Australia; 2 Department of Epidemiology and Preventive Medicine, Monash University, Melbourne, Victoria Australia; 3 Victorian Government Department of Health, Victoria, Australia; 4 School of Population and Global Health, The University of Melbourne, Parkville, Victoria Australia; 5 Department of Infectious Diseases, The Alfred and Monash University, Melbourne, Victoria Australia; 6 Department of Infectious Diseases, Doherty Institute, The University of Melbourne, Parkville, Victoria Australia

**Keywords:** COVID-19, Mathematical model, Outbreak analysis, Disease control

## Abstract

**Background:**

Policy responses to COVID-19 in Victoria, Australia over 2020–2021 have been supported by evidence generated through mathematical modelling. This study describes the design, key findings, and process for policy translation of a series of modelling studies conducted for the Victorian Department of Health COVID-19 response team during this period.

**Methods:**

An agent-based model, Covasim, was used to simulate the impact of policy interventions on COVID-19 outbreaks and epidemic waves. The model was continually adapted to enable scenario analysis of settings or policies being considered at the time (e.g. elimination of community transmission versus disease control). Model scenarios were co-designed with government, to fill evidence gaps prior to key decisions.

**Results:**

Understanding outbreak risk following incursions was critical to eliminating community COVID-19 transmission. Analyses showed risk depended on whether the first detected case was the index case, a primary contact of the index case, or a ‘mystery case’. There were benefits of early lockdown on first case detection and gradual easing of restrictions to minimise resurgence risk from undetected cases. As vaccination coverage increased and the focus shifted to controlling rather than eliminating community transmission, understanding health system demand was critical. Analyses showed that vaccines alone could not protect health systems and need to be complemented with other public health measures.

**Conclusions:**

Model evidence offered the greatest value when decisions needed to be made pre-emptively, or for questions that could not be answered with empiric data and data analysis alone. Co-designing scenarios with policy-makers ensured relevance and increased policy translation.

**Supplementary Information:**

The online version contains supplementary material available at 10.1186/s12889-023-15936-w.

## Introduction

Australia’s COVID-19 strategy has changed markedly over 2020–2022. Starting from an extended period where tight border controls, contact tracing and lockdowns were used to eliminate community COVID-19 transmission, in mid-2021 and as vaccines became available, public health restrictions were instead used to maintain health system capacity by controlling disease rather than eliminating community transmission. In the state of Victoria, Australia, the government used epidemiological modelling extensively over this period to assess the health-related outcomes of potential policies. Model outcomes were used to complement information from other sources including analysis of epidemiological data, economic forecasting, international experience and expert opinion to inform decision-making on COVID-19 policy responses.

Australia’s first wave of COVID-19 was mainly driven by imported infections from international travel [1]. All Australian States and Territories implemented lockdowns, which stopped local transmission and led to local elimination of COVID-19 (‘COVID-zero’) by June 2020 in seven of eight jurisdictions. This initial success shaped Australia’s COVID-19 pre-vaccination phase policies. Victoria was the only exception to this initial achievement of COVID-zero, experiencing an outbreak of the wild-type variant from June-November 2020. This outbreak comprised distinct periods of epidemic growth (June-August) where a sequence of public health restrictions were introduced to control it, and epidemic decline (August-November) where restrictions were incrementally eased as case numbers decreased. During this period, key policy questions centred around how to relax restrictions without epidemic resurgence. Following this outbreak, community COVID-19 transmission was eliminated from Victoria [2].

Between November 2020 and July 2021, Victoria continued to eliminate community COVID-19 transmission through international travel restrictions, hotel quarantine of international arrivals, and reactive city or state-wide lockdowns to assist intense contact tracing following incursions into the community. Relatively brief lockdowns were implemented on three occasions following such incursions. Decisions around the scale of the response for each outbreak had to be made following only a small number of detected cases and based on an incomplete understanding of how much transmission had already occurred. Key questions during this time focused on how quickly restrictions needed to be imposed, the duration of restrictions, and level of restrictions needed to contain outbreaks.

Between July 2021 and November 2021, Victoria experienced an outbreak of the more infectious Delta variant that continued to grow despite extensive lockdowns and public health measures in place. In parallel, vaccine availability was increasing, prompting a transition in COVID-19 strategy from an aim of eliminating community transmission to an aim of slowing the spread until high population-level vaccine protection was established [3]. The context of a simultaneous growing epidemic, increasing vaccine coverage and change in COVID-19 strategy required the development of a roadmap for easing restrictions. The key questions during this phase of the outbreak centred around the interaction between restrictions and vaccine coverage, and ensuring that health system demand did not exceed capacity.

By December 2021, over 90% two-dose vaccine coverage among people over 16 years had been reached in Victoria and transmission of the Delta variant appeared to be stable in the community with minimal restrictions in place [4]. Subsequently, the emergence of the Omicron variant triggered a large epidemic wave, the management of which is still ongoing.

Throughout these different epidemic stages, our modelling team worked closely with the Victorian government to conduct analyses to estimate COVID-19-related outcomes from different policy options. In this paper we present a selection of analyses that were conducted at different stages of the pandemic and describe how the modelling was used to inform decision-making at critical times. This is useful to understand the circumstances and ways in which modelling can be most valuable for policy making.

## Methods

### Model overview

We used an established agent-based microsimulation model, *Covasim* [5, 6], developed by the Institute for Disease Modelling (USA) and collaborators including the Burnet Institute, to model epidemics in Melbourne [6–8]. The model is open source and available online [9]. In brief, agents in the model are assigned an age (which affects their susceptibility to infection and disease prognosis), a household, a school (for people aged 5–17) or a workplace (for people over 18, up to 65), and they participate in a number of community activities that may include attending restaurants, pubs, places of worship, community sport, and small social gatherings. Details of included contact types, network structures, transmission dynamics and disease outcomes in the latest model version are provided in the supplement, though the model was continually adapted as analysis questions changed to enable scenario analysis of settings or policies relevant for critical decisions, and so not all features were used for all analyses presented in this paper.

### Interventions

The model includes vaccination (including individual dosing schedules, vaccine types and waning immunity), testing (PCR or rapid antigen tests), contact tracing (with probability of tracing contacts depending on the setting the contact occurs, the capacity of the system and the tracing policy at the time), quarantine of close contacts, isolation of confirmed cases, masks, and a variety of policy restrictions to prevent or reduce transmission in different settings (e.g. closing schools or workplaces, density limits in hospitality and retail settings, restrictions on social gathering sizes). Further details are provided in the supplement.

### Calibration and SARS-CoV-2 variants

Model parameters for transmission and testing were continually calibrated and adjusted to fit data on daily new detected cases, hospital demand and ICU demand for each new analysis and variant. For the wild-type variant, this was based on calibration to data over the June-November 2020 epidemic wave. For the Alpha and Kappa variants, this was based on international literature and data on the relative transmissibility and severity compared with the wild-type variant (since there were no major outbreaks in Australia). For the Delta variant this was based on calibration to data over the Aug-Sep 2021 epidemic wave.

### Scenario types

As the epidemic progressed, the types of scenarios examined changed along with the policies under consideration and key questions. Four representative scenario types were retrospectively identified for presentation in this paper (Table 1).

**Table 1:** Representative scenario types presented in this paper

Scenario type	Outcomes	Context	Calibration
Prospective outbreak	• Probability of outbreak growing under different theoretical circumstances	No community cases; elimination strategy	No parameter fitting, sensitivity analyses for key parameters
Reactive outbreak	• Probability of existing outbreak reaching different levels or being eliminated • Time required to contain outbreak	Small number of diagnosed cases following period of no community cases; elimination strategy	No parameter fitting, filter outbreaks to match observed
Easing restrictions	• Timing and magnitude of epidemic peak • Probability of resurgence following easing of restrictions	Large number of cases; elimination strategy	Fit model to cases/hospital demand/deaths
Managing health system utilization	• Timing and magnitude of epidemic peak(s) • Peak hospital/ICU demand • Number of deaths	Large number of cases; epidemic control strategy	

### Scenario type 1: prospective outbreak analyses (elimination strategy context)

The model was initialized with no cases, public health settings were varied, and one or more incursions were simulated. The model was stochastic so onward transmission did not occur in every simulation, but where transmission did occur, it could lead to case detection through symptomatic testing or surveillance testing interventions. After detection, policy options could be triggered to contain the outbreak. The main objective was to assess how different public health settings balanced the intensity and duration of restrictions against the risk of the outbreak growing out of control.

The principal output measure was the percentage of simulations where the epidemic reached different sizes over a fixed period (e.g., 90 days). Simulations were often categorized as no cases detected; some cases detected but the virus eventually eliminated; or the 7-day average daily detected cases reaching different threshold levels by the end of the model simulation.

### Scenario type 2: reactive outbreak analyses (elimination strategy context)

Outbreak analyses were calibrated to replicate outbreak characteristics at the analysis date. Simulations were sampled and rejected if they were not within +/- 10% of the cumulative detected cases. Similarly, model simulations could be filtered to include only those where particular numbers of ‘mystery cases’ (diagnosed cases for which modelled contract tracing could not find a transmission source) were detected. This filtered set of model simulations were conditioned on the observed state of outbreak so far, and were used to generate projections for the impact of prospective interventions. This meant that when an outbreak had begun with a series of ‘unlucky’ events at the start (such as chain of superspreading events), that could be captured in the model.

As with the prospective outbreak analyses, the main output measure was the percentage of simulations reaching different categories of 7-day average daily detected cases and key questions focused on the duration of restrictions required to contain the outbreak.

### Scenario type 3: easing restrictions (elimination strategy context)

For situations where a large outbreak had occurred and restrictions had already been imposed, policy questions arose about when to ease restrictions and whether to do it incrementally or collectively. Model scenarios were calibrated analogously to the constrained outbreak analyses, where the model was initialized with a small number of cases and simulations were only retained if they were consistent with the actual outbreak, accounting for restrictions imposed to date. Using only the retained simulations, scenarios were run comparing outcomes with restrictions in place for different periods.

The main outcome measure was the probability of reaching > N diagnoses per day following the easing of restrictions (i.e., “resurgence risk”), and how this varied according to the timing and extent that restrictions were eased.

### Scenario type 4: health system utilization (control strategy context)

As the broader COVID-19 strategy transitioned from elimination to control, health system utilization became increasingly relevant. Scenarios were calibrated similarly to the easing restrictions analyses, and compared the impact of dynamically introducing or easing restrictions in the context of higher case numbers. The key outcome measures were hospital and ICU demand rather than the number of cases.

## Results

**Fig. 1:**
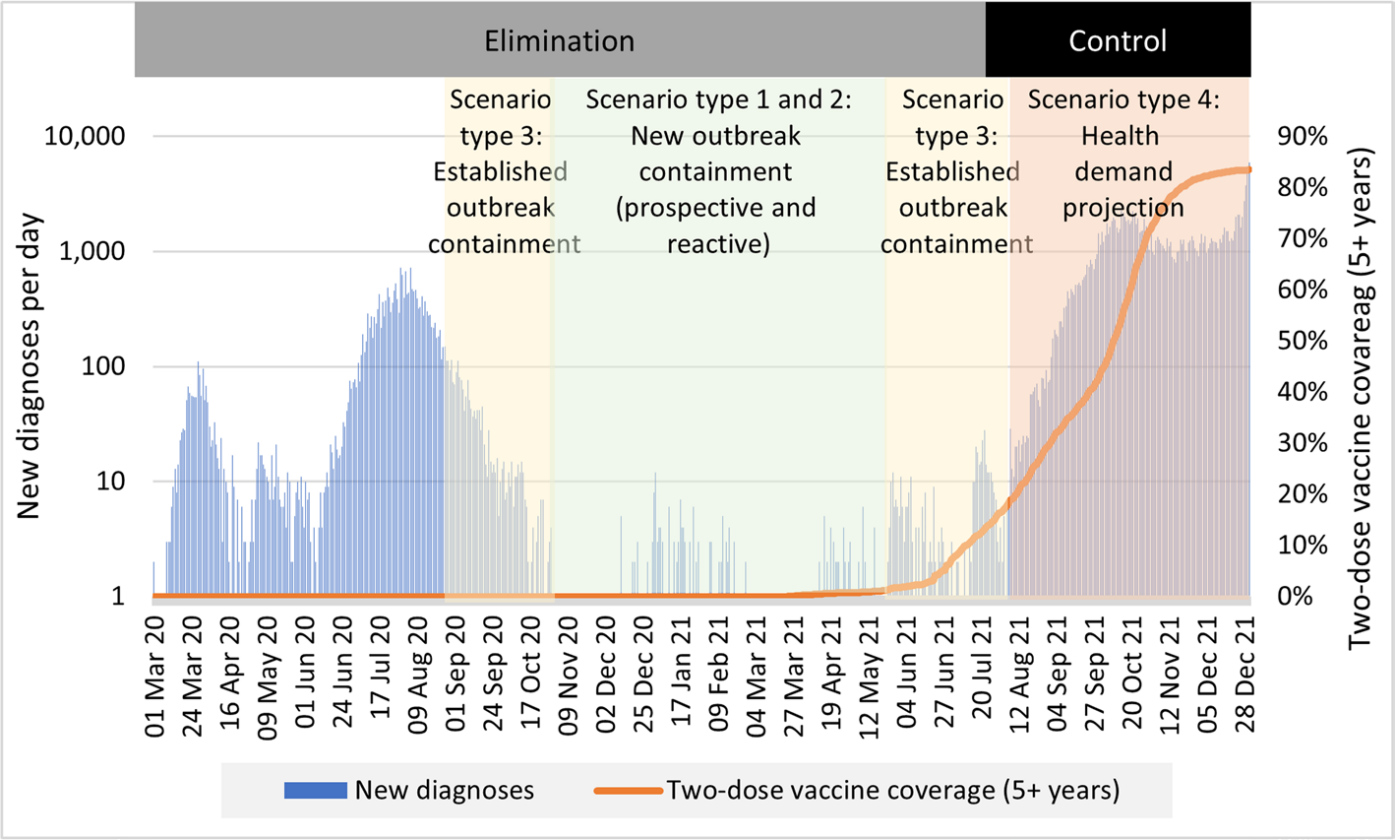
Victorian COVID-19 timeline. New daily cases and vaccine coverage are shown over time, contextualising the overall strategic phase (elimination or suppression) and the dominant scenario type being run at different stages of the pandemic (vertical shaded regions)

An overview of overarching strategy and when principal scenario types were used is shown in Fig. 1, overlaid on case numbers and vaccine coverage for epidemic context.

### Easing restrictions example: wild-type epidemic wave, leading to elimination (September 2020)

**Fig. 2:**
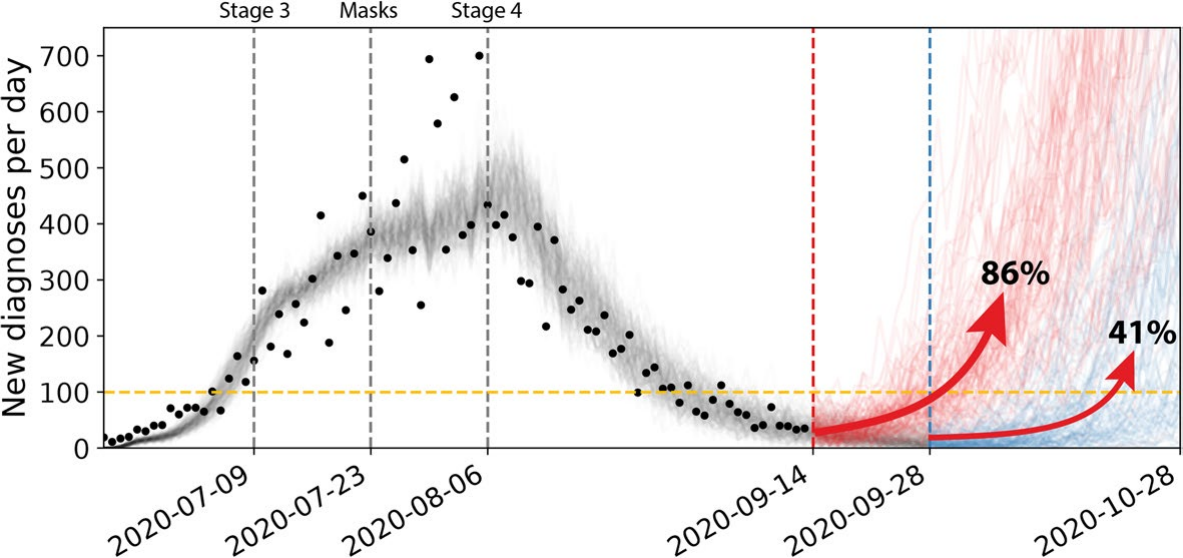
Wild-type epidemic wave in Victoria in 2020, in the context of an elimination COVID-19 strategy, with scenarios considering resurgence risk if restrictions were eased on 14 Sep (red) or 28 Sep (blue). Model simulations were started with random infected seed cases and randomly sampled transmission parameters, and were retained if they were within sufficient bounds of the observed data. Throughout the simulations ‘Stage 3’, masks and ‘Stage 4’ restrictions were imposed, with their impact derived through model calibration (see [8])

Following a wild-type epidemic wave in Victoria (June-Nov 2020) that was contained by imposing restrictions, an analysis was conducted on 14 September to assess the impact of the timing of easing restrictions on resurgence risk (Fig. 2) [8]. Model calibration involved running simulations starting from zero cases, sampling over initial seed infections (i.e. who in the model is the first case) and uncertainty ranges of calibrated transmission parameters, and retaining simulations within a threshold of the case data (Fig. 2, grey lines). For a set of 1000 retained simulations, scenarios were applied to a short-term projection to compare the impact of easing restrictions on different dates. The red and blue lines in Fig. 2 show individual simulations for each restriction scenario, highlighting the broad range of outcomes possible. For each scenario, a proportion of simulations trend to zero while a proportion result in a resurgence of infections, defined as reaching a threshold of daily diagnoses. This analysis showed that an additional two weeks of restrictions could more than halve the risk of a resurgence in cases. Factoring this and other evidence, restrictions were maintained and cases continued to decline, before being gradually eased from 28 September (e.g. small gatherings of five people outdoors).

### Prospective outbreak analysis example (October 2020)

**Fig. 3:**
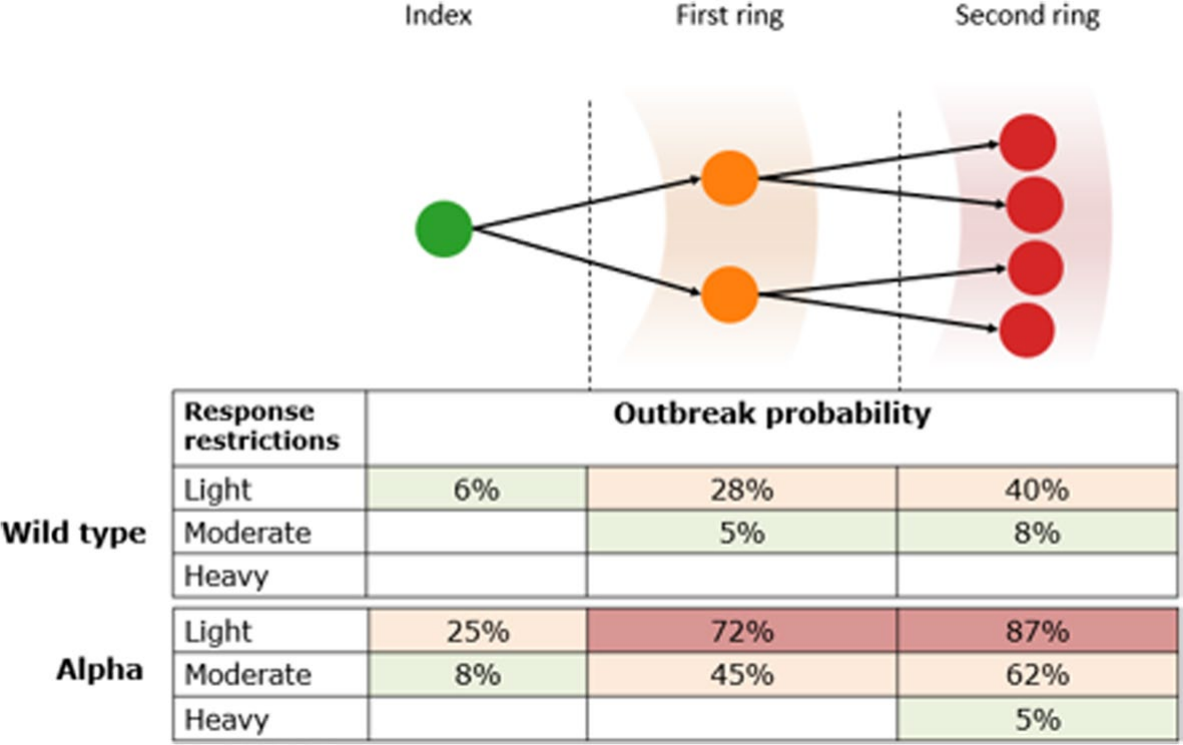
Outbreak analysis in Victoria in 2021, in the context of pursuing an elimination strategy. Simulations were run starting from zero cases and a randomly infected seed case. Table shows the proportion of simulations where an outbreak occurred (defined as reaching a 7-day average of > 30 diagnoses/day within 90 days), according to which infection generation was detected, the infectiousness of the virus (baseline = wild type; 50% more infectious ~ Alpha variant) and what responses were implemented on detection of the first case (light = masks and limits on social gatherings; moderate = light + density limits; heavy = lockdown)

After the incidence of community acquired cases returned to zero from 29 to 2020, outbreak risks from potential incursions were assessed. Simulations started with one undiagnosed case. The outbreak risk was defined as the percentage of simulations that reached a particular 7-day average diagnosis threshold within 90 days of the start of the simulation (e.g. >30 in Fig. 3). All simulations included contact tracing (including second-ring tracing for some contacts).

Several factors were identified that influenced outbreak risk including the infectiousness of the variant, the number of generations of transmissions before the first detected case, and the policy response to the initial case detection. The number of infection generations prior to the first diagnosis being recorded was particularly predictive of the outcome. In simulations where the index case was the first identified, light restriction contained the outbreak 94% of the time (Fig. 3). However, if the index case was not the first case detected, this made containment much more difficult. At the time of analysis, the Alpha variant was estimated to be 50% more infectious than the wild type variant [10, 11], and in simulations where an Alpha variant infection was identified in the community and the index case was not the first case diagnosed, then even a moderate response (mask mandates, limits on social gatherings, and density limits) would be unlikely to contain it (Fig. 3). These analyses highlighted the importance of interventions to increase case detection (e.g., asymptomatic screening of quarantine hotel workers).

**Fig. 4:**
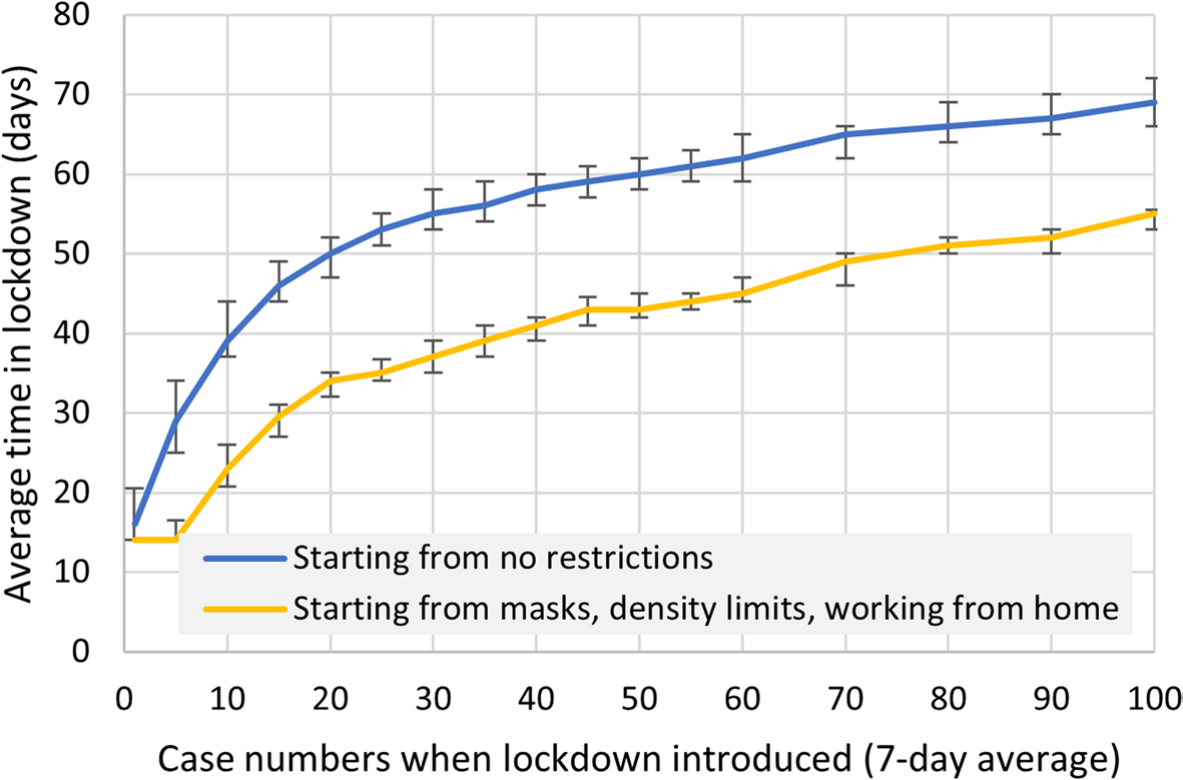
Outbreak time to containment. Following an outbreak, the time required in lockdown to return to < 5 diagnoses per day, according to the delay in implementing restrictions. Lines show median and inter-quartile range (error bars) across 1000 simulations. Based on the wild type variant

The impact of the timing of the response to an outbreak was also assessed. For early variants (wild-type, Alpha), heavy restrictions were always able to contain outbreaks, but a delay in introducing restrictions increased the duration of restrictions to contain the outbreak (Fig. 4). The analysis showed that if some restrictions were already active at the time of the incursion, the duration of restrictions would also be shorter.

This supported a “go hard go early” approach to restrictions in the elimination context, which was applied when lockdowns were imposed from 13 to 17 February 2021 after 13 Alpha variant cases were detected, from 28 May to 3 June 2021 (later extended to 10 June) after 30 Kappa variant cases were detected, and from 16 July to 20 July (later extended to 27 July) after 18 Delta variant cases were detected. After each lockdown restrictions were incrementally eased, rather than lifted at once.

### Reactive outbreak analysis example: Kappa variant outbreak (May 2021)

Once an incursion was detected, outbreak risk estimates were refined by incorporating characterisations of the first few diagnoses. In May 2021 an outbreak of the Kappa variant occurred, with the first diagnosis on 24 May, and 45 and 58 cases were diagnosed cumulatively within the next 7 and 10 days, respectively. Mandatory masks were imposed on the first day, and a lockdown was imposed on the third day.

**Fig. 5:**
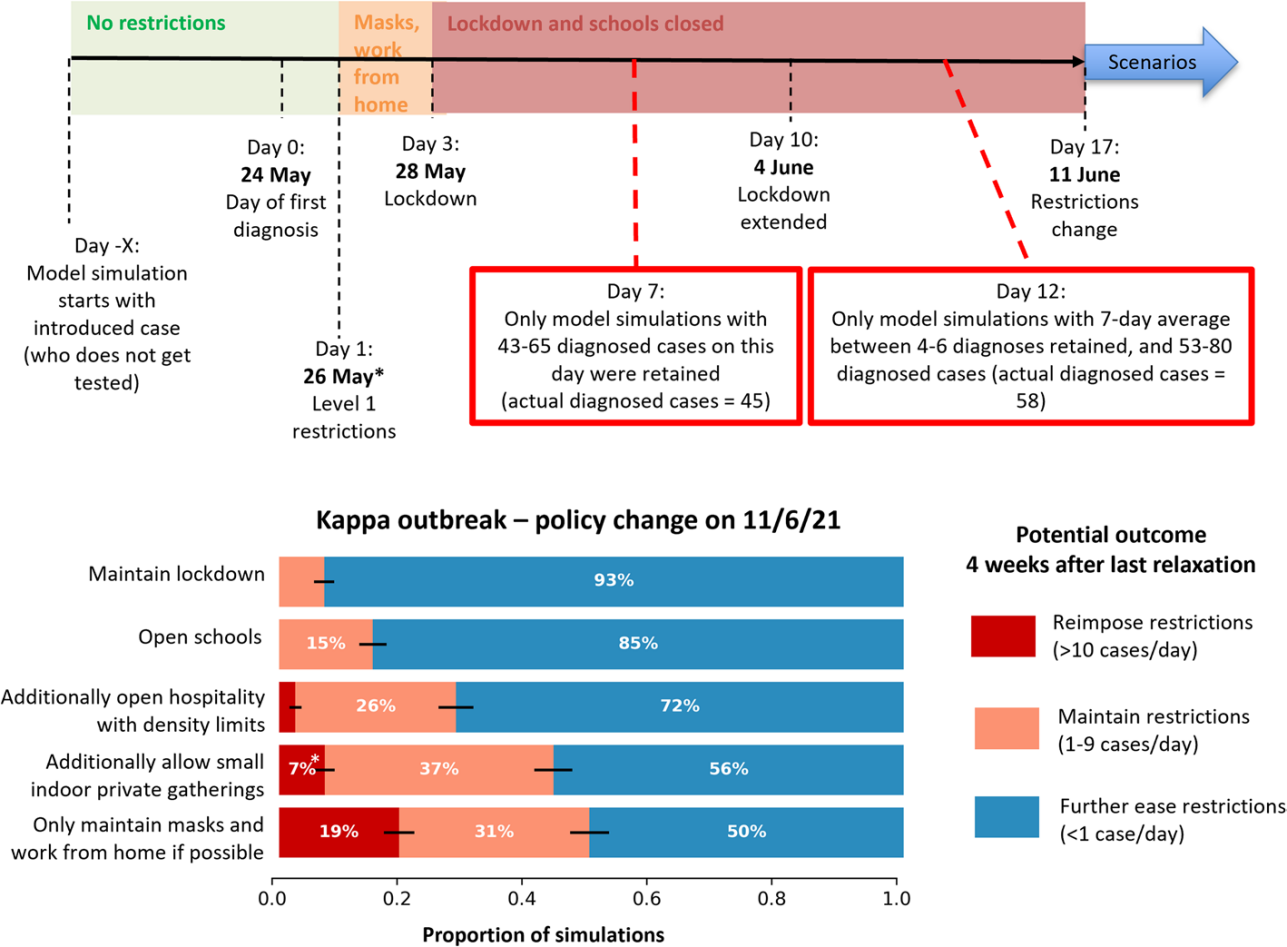
Outbreak analysis in Victoria in 2021, when an outbreak has been detected but limited information is known, pursuing an elimination strategy. Top: Simulations were run starting from zero cases and a randomly infected seed case. Simulations were only retained if they produced, through stochastic variation, approximately the same initial outbreaks as occurred. In this example it was 43–65 diagnoses within 7 days of the first diagnosis, and 53–80 diagnoses within 12 days of the first diagnosis. This is against a background of masks and work from home if possible being implemented on day 1, and lockdown being implemented on day 3. Bottom: From the retained simulations, scenarios compared whether the lockdown was maintained, or after 14 days either schools were reopened, venues were opened with density limits, small social gatherings were also allowed, or a return to masks and working from home only

Modelling was used to estimate the duration of the lockdown needed to contain the outbreak. Simulations started with a single case and were retained if transmission occurred (i.e., the incursion did not fizzle out), the index case was never diagnosed, and there were between 43 and 65 diagnoses after 7 days and 53–80 diagnoses after 12 days. Using 1000 simulations that met these criteria (sampling over initial seeds, contact network structures and transmission parameters), scenarios were run for policy changes under consideration, where after two weeks of lockdown (June 11), either the lockdown was maintained, or restrictions were eased by: opening schools only; opening schools and venues with density limit; opening schools, venues with density limits and allowing small social gatherings; or returning to masks only. The impact of these policy changes on daily diagnoses four weeks after implementation was recorded as the main outcome measure (Fig. 5). The analysis quantified how much the risk of a resurgence increased when restrictions were eased more generously, and enabled resurgence risk to be incorporated into decision-making alongside the costs associated with the lockdown.

Based on this and other evidence, from 11 June schools were re-opened, hospitality was reopened with density limits, small gatherings were allowed but only outdoors, and work from home and mask mandates were maintained.

### Prospective outbreak analysis example: context of vaccine rollout and coverage (March 2021)

**Fig. 6:**
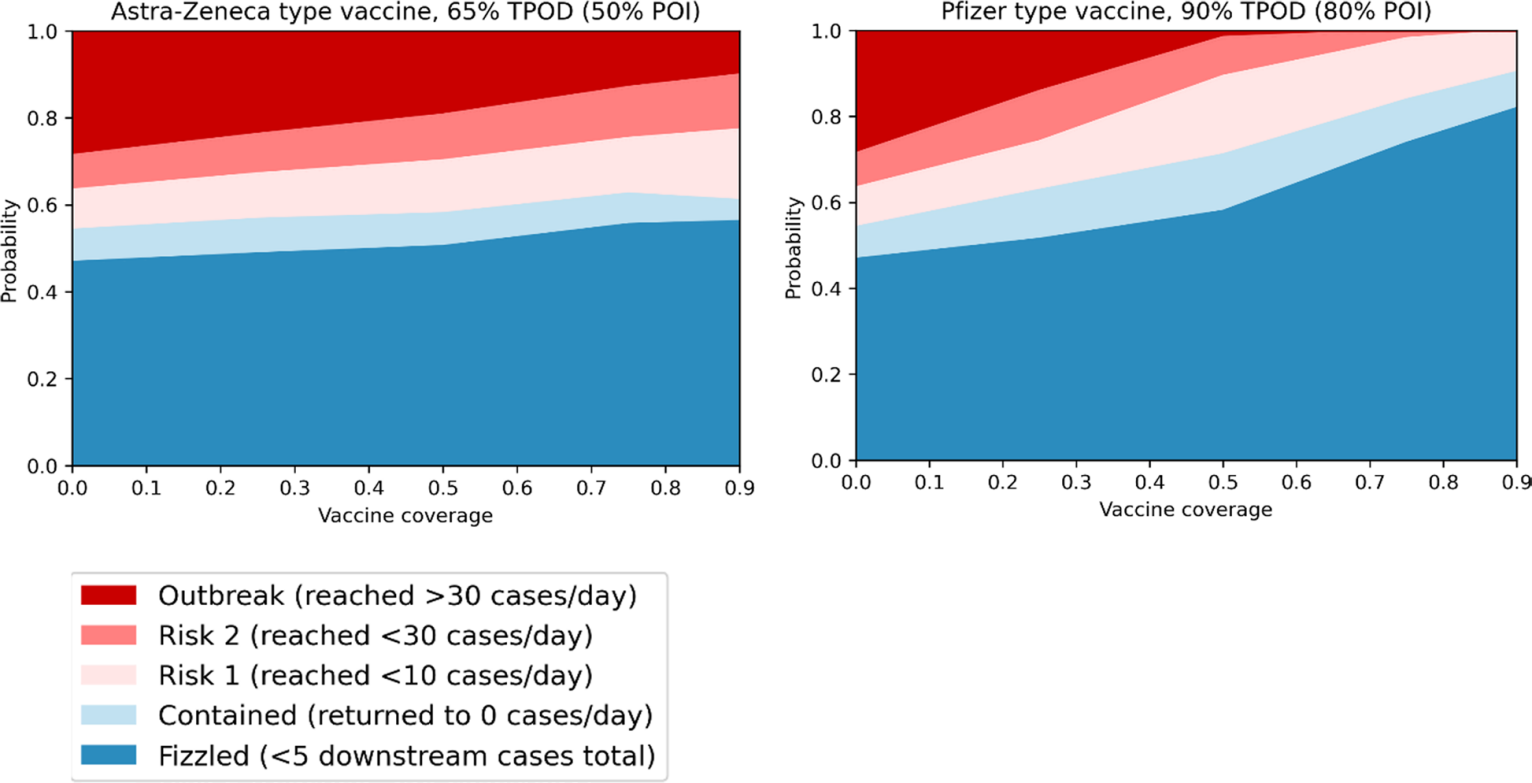
Outbreak analysis in Victoria in 2021, over 90 days starting from a single case and different levels of population vaccine coverage. Left: AstraZeneca type vaccine, with assumed protection against infection of 50%, and protection against symptomatic disease of 65%. Right: Pfizer type vaccines, with assumed protection against infection of 80%, and protection against symptomatic disease of 90%. TPOD = total protection against disease; POI = protection against infection. Note that vaccine efficacy estimates were based on best available data as at March 2021

When vaccination coverage increased, other public health responses were also expected to change. In the context of vaccines becoming available, outbreak risk (defined as the percentage of simulations reaching different diagnosis thresholds over the first 90 days after a single case) was assessed by vaccine type and coverage (Fig. 6). While outbreak risk reduced with increasing vaccine coverage, it was clear that herd immunity through vaccination was unlikely to be achieved, supporting an eventual move from an elimination strategy to a control strategy.

### Managing health system utilization example: moving from elimination to control (July 2021)

**Fig. 7:**
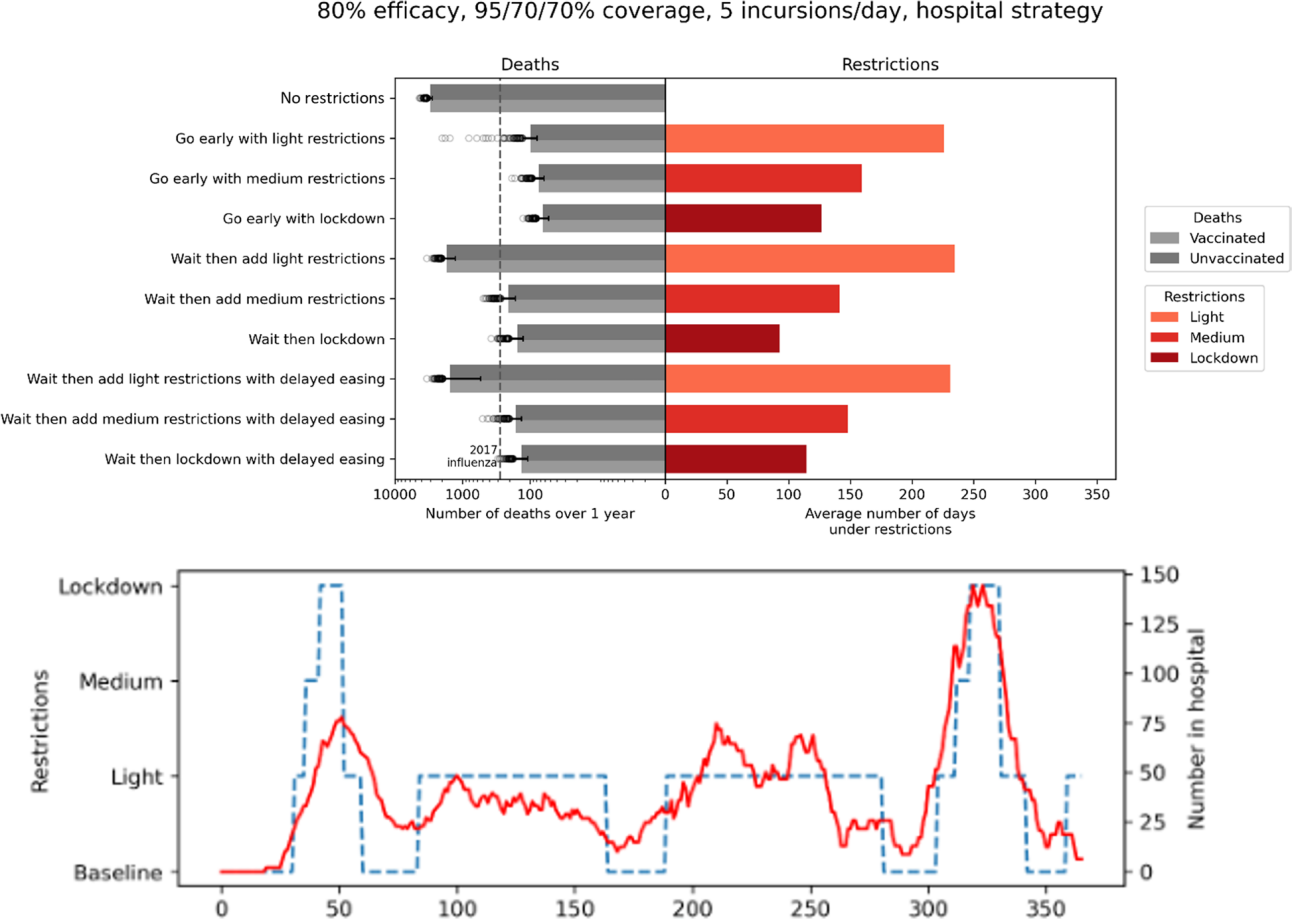
Projected COVID-19 deaths in Victoria over a 12-month period under different outbreak response scenarios, triggered by case numbers (left), and average time spent under restrictions (right). Top: Grey bars show the projected number of deaths on a log scale, with the lighter shading indicating the proportion that are among the vaccinated population. Scenarios are based on 95%/70%/70% vaccine coverage being achieved among people aged 60+/12–59/<12 years, the vaccine having an assumed 80% protection against infection and 92% protection against death, and 5 cases per day were seeded into the community through reduced quarantine measures. Light restrictions = mandatory masks, density limits and work from home if possible; medium restriction = light restrictions + additional limits on gathering sizes; lockdown = mandatory masks, work from home, schools closed, retail closed, hospitality take-away only, social gatherings up to two outdoors only. Bottom: example time series output from one simulation, where increasing restrictions are triggered with increased hospital number thresholds, to maintain disease control

Later we investigated the feasibility of a range of possible control strategies, from intermittent restrictions to removal of all non-pharmaceutical interventions (NPIs) [12, 13]. Since vaccines were highly efficacious but imperfect, removing all NPIs was found to come at considerable health cost (Fig. 7). This analysis showed that with the Delta variant, intermittent low-level restrictions (for example, high levels of testing, mask mandates and work from home) could potentially reduce COVID-19-related mortality to a similar rate as experienced in the 2017 influenza season (a year with particularly high influenza-related mortality), depending on vaccine efficacy. In addition, a trade-off was identified between the degree of restrictions and the duration, where similar case numbers could be achieved with a short duration of strict restrictions, or longer duration of light restrictions. This showed that even with high vaccine coverage, without NPIs the health impacts of COVID-19 would be much worse than influenza, but that intermittent light restrictions could considerably improve outcomes.

### Managing health system utilization example: Delta variant epidemic wave (Sept 2021)

**Fig. 8:**
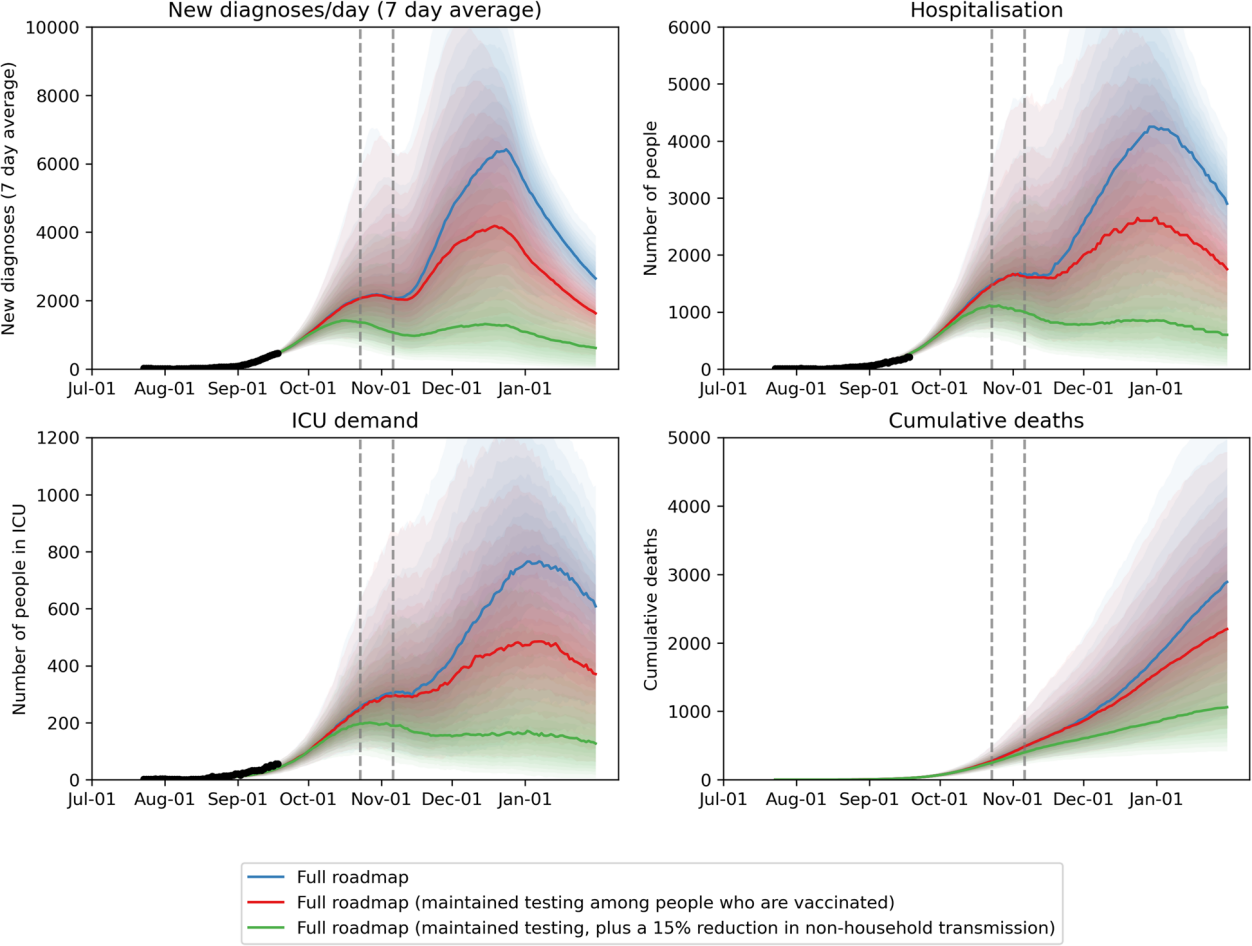
2021 roadmap with reduced testing for people vaccinated (blue), maintained testing for people vaccinated (red), and an additional 15% reduction in non-household transmission (green). Scenarios include schools returning to in person learning throughout October; childcare returning and mobility restrictions easing in October; limited outdoor gatherings at 70% two-dose vaccine coverage among people 16 + years; indoor gathering with density limits at 80% two-dose coverage among people 16 + years and mandatory vaccine requirements. Dashed vertical lines represent estimated dates of reaching 70% and 80% two-dose coverage among people 16 + years

Prior to the Delta variant, NPIs were sufficient to contain outbreaks in unvaccinated populations. However, in mid-2021 Victoria experienced a large Delta variant epidemic wave before high coverage of vaccination was achieved (as of 5 August only 43% of Victorians over 16 years had received one dose and 21% two doses). With the Delta variant having a shorter serial interval that reduced the effectiveness of contact tracing, and community fatigue leading to declining compliance with restrictions, community transmission continued to increase despite a lockdown being imposed. Plans to transition away from an elimination approach were accelerated, with a focus on using NPIs to control the epidemic while conducting a mass vaccination campaign. Modelling was used to assess the implications of different strategies (i.e., sequences and timings of restrictions being eased, relative to the vaccination rollout and case numbers) on infections and health system demand [14, 15]. This analysis showed that maintaining testing among those vaccinated was likely to be necessary for limiting transmission, and early actions to achieve transmission reduction could have significant downstream implications due to rising vaccine coverage (Fig. 8). The roadmap that was developed ultimately included incremental easing of restrictions in line with different vaccination coverage thresholds.

## Discussion

After Australia’s initial response successfully eliminated community COVID-19 transmission from most jurisdictions in early 2020, and as the dire health consequences of widespread transmission in the absence of a vaccine were observed internationally, Australia embarked on a phase lasting until mid-2021 where similar catastrophic health outcomes were avoided by using tight border controls, contact tracing and restrictions to achieve and maintain the elimination of community transmission. During this phase, community incursions from hotel quarantine regularly occurred, and modelling was used as a critical tool to understand outbreak risks and how they varied in different circumstances or with different responses.

In the elimination phase, model outputs showed outbreak risk depended on whether the detected case was the incursion, first ring of infections or a ‘mystery case’. There were benefits of early lockdown on detection of the first case and exiting lockdowns slowly through gradual easing of restrictions to minimise resurgence risk from undetected cases. Key metrics in this period were the number of cases, the intensity of restrictions, and the duration of restrictions, as returning to COVID-zero was viewed as the most efficient way to meet other targets.

The emergence of COVID-19 vaccines meant that catastrophic health outcomes could be avoided without the need for some of the stricter border control and lockdown measures. This, combined with the fact that the elimination of community transmission was no longer realistic due to the emergence of more infectious variants, led to a move from an elimination to a control strategy. While high vaccine coverage was an essential element, our analyses indicated that sustainable control strategies would require vaccines plus intermittent ‘light’ restrictions, introduced early in times of case number escalation, to avoid excess case and health system demand escalation. During the critical transition period from an elimination to a control strategy, and as vaccine coverage increased, the focus of analyses shifted to health system demand, with case numbers becoming less important.

In all analyses conducted, model evidence was most crucial for decision making when used to investigate questions that could not be answered in any other way. For example, where relevant data was available, direct analysis was significantly faster to inform decisions and required fewer assumptions to be made. Model analyses were therefore typically used to support decisions that needed to be made pre-emptively (e.g., how long to lockdown for, when minimal information was available) [16]. Since the primary aims of each study could not be answered directly with data, this meant that the rationale for the use of modelling was always clear.

The utility of modelling was also increased by the co-design of scenarios with government and other relevant stakeholders [16]. Scenarios were only modelled if (a) they could not be shown to be superior / inferior based on existing data; (b) they were considered reasonable by epidemiologists and public health teams; and (c) they were being seriously considered by government given other economic, social, and political implications. Many policy options could be ruled out before even making it to the modelling stage, because they were not likely to be feasible for other reasons. For example, working with stakeholders ensured that scenarios could incorporate operational or capacity constraints in the delivery of services such as testing or vaccination. Co-designing the scenarios also helped align model outputs with other evidence under consideration, to make the modelling as informative as possible. For example, running epidemic projections for similar scenarios to those separately used for economic forecasts facilitates integrating both sources of evidence. In addition, the continued involvement of the modelling team with public health teams allowed ongoing refinement and improvement of the modelling, and validation against past accuracy.

There were many challenges associated with the need for fast decision-making. Ideally, modelling analyses would undergo peer review before being used to inform decisions. The purpose of the peer-review process is to ensure that model design, assumptions and inputs are fit-for-purpose, consistent with best practice, and incorporate the latest evidence. However, in the case of an outbreak, decisions often need to be made quickly, with significant impacts on the lives of large numbers of people. It is therefore impractical to have outputs peer-reviewed in advance, which can take months. Review and validation of raw model outputs by government analysts on standby was conducted in place of such checks, and where feasible identical requests were often put to other modelling groups [17, 18] to be conducted simultaneously and independently [19, 20].

Detailing how these analyses were conducted is important because the role of modelling in informing policy is not well understood. Epidemiological modelling is a specialized discipline that experienced increased media attention because of its utility to generate evidence to support decision-making in the COVID-19 pandemic; however, it is often not communicated well through the media [19]. There are common misconceptions about the differences between forecasting (trying to predict the specific course of the epidemic) and scenario analyses (comparing projections under different policy options to estimate the impact of policies and inform decisions) [21]. This often results in a dichotomisation of modelling being viewed by the public as being right or wrong, with many of the key insights generated by the analyses being missed. This is compounded by the presence of uncertainty at multiple levels, including in data, model parameters, and stochastic model outputs. Communicating uncertainty and confidence is challenging, but critical to interpreting model findings. A strength of modelling that can be difficult to communicate is that even in the presence of high uncertainty in forward epidemic projections, there may be low uncertainty in what is a superior intervention or policy option [21].

There are many limitations in the use of modelling to inform COVID-19 decisions. First, models are simplifications of the real world and cannot capture the full range of human behaviour. Second, there are many things that are unknown about COVID-19 disease dynamics, that models must approximate with the evolving results of clinical trials and observational studies. Third, parameters continue to change over time. As evidence for model inputs like vaccine efficacy or human behaviour become clearer over time as more data becomes available, results change and this can make analyses done at different times appear to be contradictory. Fourth, compromises are required to constrain levels of detail in order to meet timelines for when decisions need to be made. Finally, epidemiological models typically only focus on health outcomes (and sometimes health-related costs). Government decisions have repercussions throughout the community for a wide range of stakeholders, and policy decisions must necessarily account for factors not included in the model. It is important that model analyses acknowledge this and avoid making policy recommendations outside of their ability to meet specific health targets. Overall, greater transparency around modelling and how it was used would help to educate the community, media and governments more broadly on the strengths and limitations of these analyses, and the ways in which modelling is (and is not) used in decision making.

## Conclusion

Model evidence offers the greatest value to support COVID-19 decision making when decisions need to be made pre-emptively, or to answer questions that cannot be answered with data analysis alone. Models need to adapt to evolving COVID-19 strategies and should have scenarios and outcome metrics co-designed with policy-makers and other stakeholders. Epidemiological models cannot cover all outcomes from policy changes, particularly when timelines for decision-making are short, and must be used in conjunction with other information sources to make policy decisions.

## Supplementary Information

**Additional file 1.**

## Data Availability

This study involved the use of an agent-based model, and input parameters for the model are available in the supplementary materials. The model code is available from https://github.com/InstituteforDiseaseModeling/covasim.
