# Supplementary material for: COVID-19 epidemic modelling for policy decision support in Victoria, Australia 2020–2021
Source: BMC Public Health. 2023 May 27;23:988. doi: 10.1186/s12889-023-15936-w (PMC10219801; doi:10.1186/s12889-023-15936-w)
Supplement: Supplementary file 1 — Additional file 1. [file 12889_2023_15936_MOESM1_ESM.pdf]

Supplement

The agent-based model Covasim simulates the spread of SARS-CoV-2 among a collection of agents representing people. Each agent is characterised by a set of demographic and disease properties:

- Demographics:
    - Age (one-year brackets)
    - Household size, and uniquely identified household members
    - Uniquely identified school contacts (for people aged 5-18)
    - Uniquely identified work contacts (for people aged 18-65)
    - Average number of daily community contacts (multiple settings / contact networks modelled, described below)
- Disease properties:
    - Infection status (susceptible, exposed, recovered or dead)
    - Whether they are infectious (no, yes)
    - Whether they are symptomatic (no, mild, severe, critical; with probability of being symptomatic increasing with age, and the probability of symptoms being more severe increasing with age)
    - Diagnostic status (undiagnosed, diagnosed)
    - Level of neutralizing antibodies (see vaccine section)

Transmission is modelled to occur when a susceptible individual is in contact with an infectious individual through one of their contact networks. The probability of transmission per contact is calibrated to match the epidemic dynamics observed and is weighted according to whether the infectious individual has symptoms, the type of contact (e.g. household contacts are more likely to result in transmission than community contacts), and the level of vaccine or exposure-acquired protection that the susceptible person has.

Model population

A synthetic model population is initialized comprising of 100,000 people. The age and household size structure of the model population is based on the Victorian population.

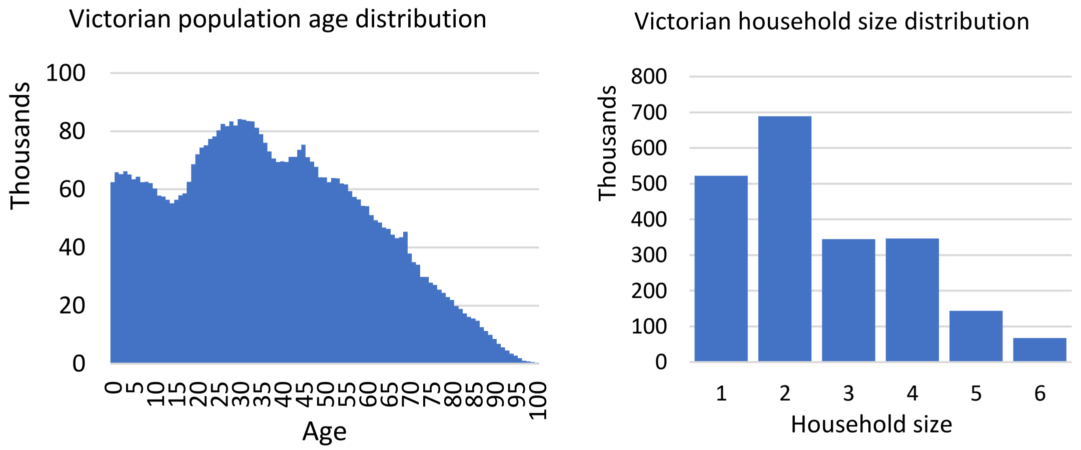

Figure S1: Population age structure and household size distribution, March 2021 estimates from Australian Bureau of Statistics [1, 2].

Household contact network: household size and age structure

The household contact network was set up by explicitly modelling households. The households size distribution for Australia [2] was scaled to the number required for the number of agents in the simulation. Each person in the model was uniquely allocated to a household. To assign ages, a single person was selected from each household as an index, whose age was randomly sampled from the distribution of ages of the Household Reference Person Indicator in the 2016 Census [2]. The age of additional household members were then assigned according to Australian age-specific household contact estimates, by drawing the age of the remaining members from a probability distribution based on the row corresponding to the age of the index member.

Primary school contact networks

Over time, the complexity of the schools networks in the model have increased.

In the model's latest implementation, primary schools are modelled as a collection of classrooms, aggregated into schools. Each student is assigned to a classroom with others of the same age, and each classroom has an assigned teacher (Figure S2). Primary school mixing includes student-student contacts within classrooms, student-student contacts between students in different classrooms, teacher-teacher contacts and teacher-student contacts within the classrooms that they are assigned to.

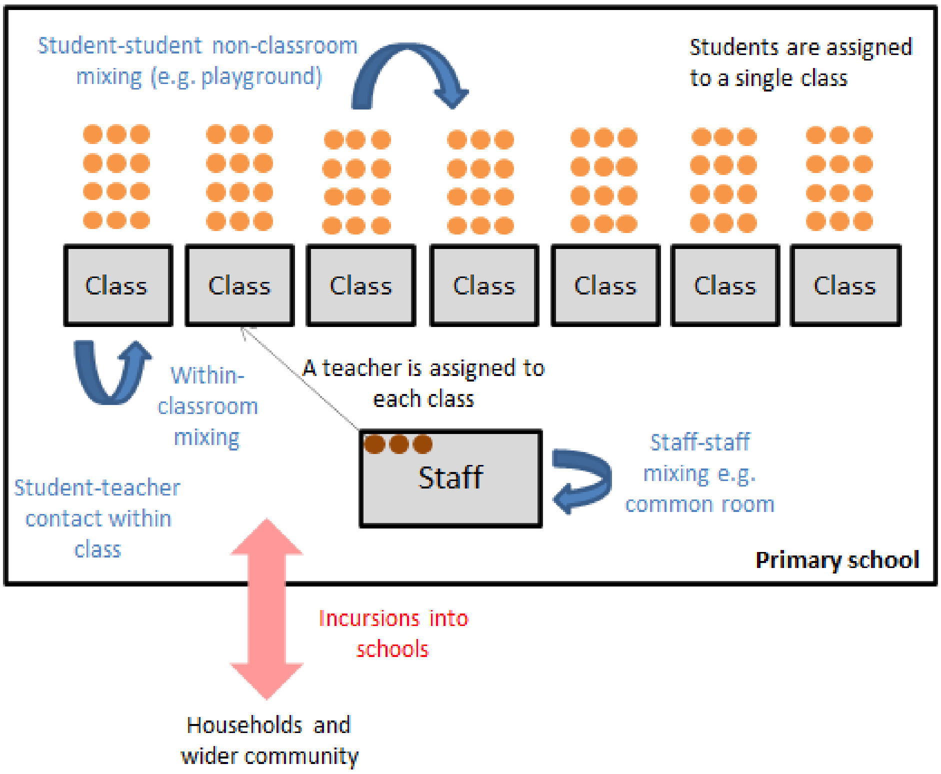

Figure S2: Contact networks within primary schools in the model. Primary schools are modelled as a collection of classrooms, where students of the same age are assigned a teacher. Primary schools include student-student

classroom contacts, student-student non-classroom contacts, teacher-teacher contacts and teacher-student contacts.

Secondary school contact networks

Secondary schools are modelled with a lower emphasis on assigned classrooms reflecting elective subjects, and hence secondary school students have a greater number of classroom contacts than primary school students. Secondary schools in the model include student-student classroom contacts, student-student non-classroom contacts, teacher-teacher contacts and teacher-student contacts (Figure S3).

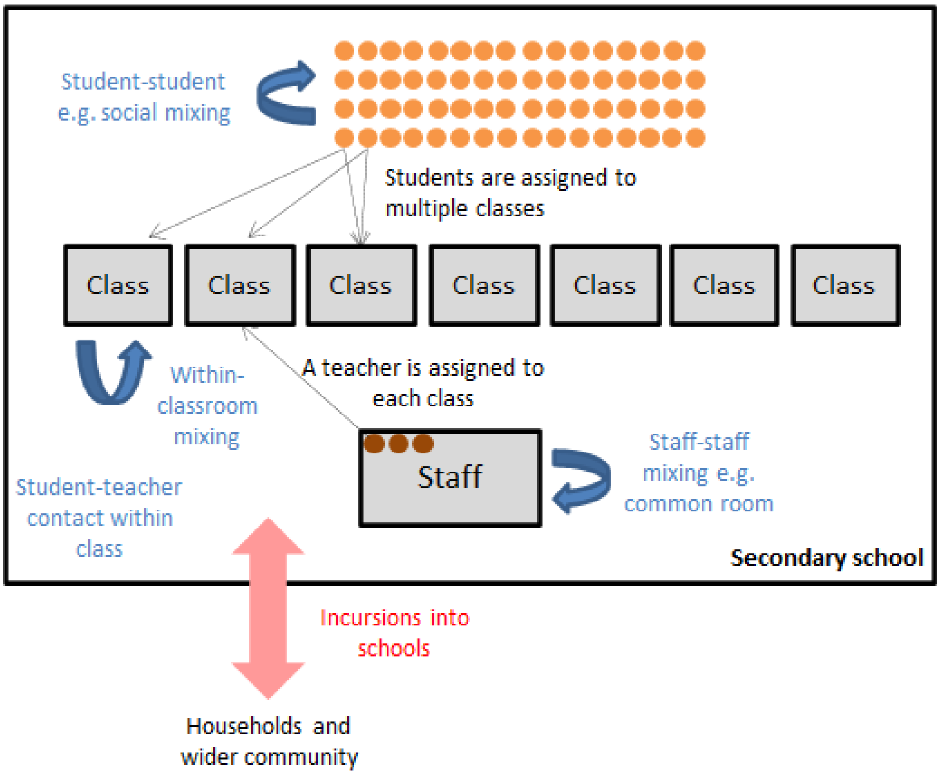

Figure S3: Contact networks within secondary schools in the model. Secondary school mixing includes student-student classroom contacts, student-student non-classroom contacts, student-teacher contacts, and teacher-teacher contacts. Secondary school students have more contacts than primary school students because they attend multiple classes.

Table S1: Model parameters related to schools

Parameter area	Estimate	Source
Primary school		
Average school size	298	Number of primary students (2,267,564 in 2020; ABS [3] Table 42b) divided by number of Primary + Primary/secondary schools (6249+1363 in 2021; ABS [3] Table 35b).
Average class size	22	Average class size of primary schools. Victorian government [4]
Average number of student-student non-classroom contacts per day, per student	2	Assumption; tested in sensitivity analysis. This impacts the efficacy of test-to-stay of class contacts verses close contacts or entire school.

Average number of teacher-teacher contacts per day, per teacher	20	Number of FTE primary teachers (152,281 in 2020; ABS [3]) divided by number of primary schools (6249+1363)
Secondary school		
Average students per school	622	Number of secondary students (1,738,083 in 2020; ABS [3] Table 42b) divided by number of Secondary + Primary/secondary schools (1433+1363; [3] Table 35b)
Average teacher/student ratio	12	ABS data. [3] suggesting secondary schools have on average 12.1 students to one teacher.
Average number of student-student classroom contacts per day	44	Average class size in secondary school of 22 ([5]; page 354), assuming two unique classrooms of contacts per student per day.
Average number of student-student non-classroom contacts per day	5	Assumption; tested in sensitivity analysis. This impacts the efficacy of test-to-stay of class contacts verses close contacts or entire school.
Average number of teacher-teacher contacts per day	5	Assumption.
Average number of teacher-student contacts per day, per student	6	Assumes students have six classes per day
Probability of transmission per contact per day (without vaccines or NPIs)		
Student-student (primary classroom)	0.25	Delphi process; Scott et al. [6] Measured as relative to household transmission per contact - e.g. a typical day's worth of contact in school is 75% less likely to result in transmission than a typical day's worth of contact at home. Non-classroom primary school contacts equivalent to outdoor contacts; secondary school classroom contacts halved to account for shorter interactions. All transmission probabilities are scaled in sensitivity analyses when NPI efficacy is tested.
Student-student (primary non-classroom)	0.03	
Student-student (secondary class contact)	0.12	
Student-student (secondary close/social contact)	0.12	
Teacher-teacher	0.25	Assumption that transmission risks in schools are equivalent for all types of contacts. Note that the model has independent parameters to account for differences in susceptibility by age
Teacher-student (primary)	0.25	
Teacher-student (secondary)	0.12	

<u>Work contact networks</u>

Within the model, people aged 18-65 years were classified according to:

- Whether they were working or not (69% classified as working, based on ABS employment data)
- Industry type, with the workforce classified as:
    - hospitality or entertainment (9% of workers);
    - retail (11%); and
    - other non-retail workers (80%; including 7% construction).

Two different workplace types are included based on the above classification: public facing (e.g. retail, hospitality) and non-public facing. Contact networks for non-public facing workplaces are created as a collection of disjoint, completely connected clusters for the percentage of people aged 18-65 who worked in those settings. The mean size of each cluster is equal to the estimated average number of daily work contacts (Table S2). For the percentage of people aged 18-65 who worked in public facing workplaces, their workplace networks consist of a completely connected cluster with other work colleagues, as well as each day having a number of random contacts with the community.

Additional contact networks

An arbitrary number of additional networks can be added. Each network layer requires inputs for: the proportion of the population who undertake these activities; the average number of contacts per day associated with these activities; the risk of transmission relative to a household contact (scaled to account for (in)frequency of some activities such as pubs/bars once per week); relevant age range; type of network structure (random, clustered, or specialized [as per schools/workplaces]); and effectiveness of quarantine and contact tracing interventions. Parameters for the networks currently in the model are in Table S2.

Parameter values for each contact network

Table S2 shows the parameters that define each contact network in the model. Unless otherwise noted, parameters are derived in [6] from a mix of published and grey literature and a Delphi parameter estimation process. The columns refer to:

- **Network structure type:** Clustered refers to a network structure comprised of disjoint, completely connected groups of contacts. Random refers to individuals being allocated connections to anyone else in the network. Random networks are also dynamic and regenerated each day. Public facing networks are a combination of completely connected clusters for staff, who are then connected to random community members
- **Mean contacts:** The average number of contacts per person in each network. Each person in the model has their individual number of contacts draw at random from a Poisson distribution with these values as the mean. For the social network layer, a negative binomial distribution was used with dispersion parameter 2 to account for a longer tail to the distribution.
- **Mean public-public contacts:** For the percentage of people who participate in an activity, the average number of contacts they have with other members of the public (draw at random from a Poisson distribution with these values as the mean)
- **Mean public-staff contacts:** For the percentage of people who participate in an activity, the average number of contacts they have with staff (draw at random from a Poisson distribution with these values as the mean)
- **Relative transmission risk:** The transmission probability per contact is expressed relative to household contacts, and reflects the risk of transmission depending on behaviour. For example, a casual contact in a public park is less likely to result in a transmission event compared to a contact on public transport. Similarly, the relative transmission risks between staff-staff, public-public and staff-public are characterised for public-facing workplaces.
- **Percentage of population:** Each network will only include a subset of the population e.g. every person has a household, but not every person regularly uses public transport.
- **Age bound:** Each network will only include agents whose age is within this range.

Table S2: Contact parameters for each of the networks in the model.

Contact network	Network structure type*	Mean contacts	Mean public-public contacts	Mean public-staff contacts	% of workforce	Relative transmission risk	Relative transmission risk (staff-staff)	Relative transmission risk (public-public)	Relative transmission risk (staff-public)	% of population	Age bound
House	Specialized	4				1.00					
School	Specialized										5-17
Non-retail work	Specialized	5			0.80	0.28					
Retail work	Public facing	5	8	2	0.11		0.28	0.04	0.04	0.70	12+
Community (general)	Random	1				0.10				1.00	
Places of worship	Clustered	20				0.04				0.11	
Community sport	Clustered	30				0.07				0.34	4-30
Entertainment	Public facing	25	8	2	0.02		0.28	0.01	0.01	0.30	15+
Cafe/restaurant	Public facing	5	8	2	0.02		0.28	0.04	0.04	0.60	12+
Pub/bar	Public facing	5	8	2	0.03		0.28	0.06	0.06	0.40	18+
Public transport	Random	25				0.16				0.11	15+
Public parks	Random	10				0.03				0.60	
Child care	Clustered	20				0.25#				0.55	1-6
Social	Random	6 (disp=2)				0.12				1.00	15+
Aged care	Clustered	12				0.58				0.07	65+

Contact tracing

Following detection of a positive case, the model initiates a contact tracing algorithm, which varies depending on tracing policies at the time, potential capacity constraints, and different algorithms for people who are identified (i.e. duration of quarantine and testing requirements). The basic algorithm is as follows:

1. Day 0: Test is taken by index case
2. Day 1 (24 hours following test^): Positive test results are returned, index case is notified and enters isolation.
3. Day 2 (48 hours following test being taken^): Contact tracing completed, with contacts having a setting-specific probability of being detected (Table S3), reflecting differences in the level of difficulty in identifying contacts in that network (e.g. households vs public transport contacts), or policies that exempt settings from tracing. Specific requirements are put in place for identified contacts, that can include different durations of quarantine or requirements to test. Tracing can take place at the individual or household level (i.e. if a contact is identified and required to quarantine, their household may need to quarantine as well).
4. Day 3 (72 hours following test^): Test results for identified contacts become available, and any contacts who returns a positive initial test would then have their contacts traced within the next 24 hours, in the same manner as the index case.

^Test return time is modelled to deteriorate as testing volume increases, based on Victorian Department of Health data. Capacity constraints can be applied for particular settings as case numbers increase; however they do not apply to household, school or childcare contacts who are assumed able to conduct their own tracing.

Table S3 shows parameters that define the contact tracing through each network in the model.

- **Contact tracing probability:** Probability that each contact can be identified in order to quarantine. This has changed over time depending on the COVID-19 strategy (elimination versus suppression) and capacity of the tracing system.
- **Effectiveness of quarantine and isolation:** When a close contact is asked to quarantine for 14 days, or a confirmed case asked to isolate while they are infected, these parameters represent the effectiveness of at reducing transmission through the specific networks. For example quarantine is assumed to have no impact on household transmission and greater impact on other contacts, reflecting compliance.

Table S3: Contact tracing parameters for each of the networks in the model.

Contact network	Assumed contact tracing probability (low cases)^	Assumed contact tracing probability (high cases)#	Assumed self-tracing probabilities&	Assumed contact tracing probabilities during Omicron outbreak*	Assumed effectiveness of quarantine on network	Assumed effectiveness of isolation on network
House	0.9	0.9	0.9	0.9	0.00	0.80
School	0.9	0.9	0.72	0	0.99	0.99
Non-retail work	0.5	0.2	0.4	0	0.90	0.90
Retail work	0.5	0	0	0	0.90	0.90
Community (general)	0.1	0	0	0	0.80	0.80
Places of worship	0.5	0	0.15	0	0.99	0.99
Community sport	0.5	0	0.15	0	1.00	1.00
Entertainment	0.5	0	0.15	0	1.00	1.00
Cafe/restaurant	0.5	0	0.15	0	1.00	1.00
Pub/bar	0.5	0	0.15	0	1.00	1.00
Public transport	0.1	0	0	0	0.99	0.99
Public parks	0.1	0	0	0	1.00	1.00
Child care	0.9	0.9	0.72	0.5	0.99	0.99
Social	0.4	0.2	0.32	0.25	0.50	0.80
Aged care	0.9	0.9	0.90	0.9	0.80	0.80

^Assuming extensive interview with Department of Health, in the context of elimination
In the context of elimination, but with high case numbers such that capacity is limited and prioritized. As case numbers increase, tracing probabilities are extrapolated between the low case and high case number limits, reaching the high case number limits at 500 cases per day.
& Assuming no input from Department of Health, and so probabilities are scalable with case numbers. Requires information systems to be accessible for people who test positive.
* Following the changed definition, from 1 Jan 2022, that close contacts are household contact or people who have spent more than 4 hours in a household-like context together.

Testing

All people with severe disease are assumed to be tested. For people with mild symptoms, the model includes a per-day probability of seeking a test, which is determined through model calibration. As rapid antigen tests have become available, the model includes a per-day probability of seeking a RAT as well.

The model also includes a per-day probability of asymptomatic people seeking either a PCR test or a RAT in more recent analyses (i.e. people without COVID-19, as well as people who have an asymptomatic infection). The probability of asymptomatic PCR testing is calibrated to match the total number of tests processed. Asymptomatic RAT testing can be implemented at different rates for different sub-populations; for example teachers could be modelled to test twice weekly as part of a surveillance program.

Table S4: Test sensitivity parameters.

Test sensitivity		
PCR test sensitivity	0.87	Arevalo-Rodriquez et al. systematic review [7]
Rapid antigen test sensitivity	0.773	Muhi et al. [8] Lower bound selected to account for inconsistent self-use. Note that PCR is modelled as having 87% sensitivity in real world use (systematic review Arevalo-Rodriguez et al. [7])

Vaccines and immunity

In the model, vaccination acts to reduce the probability of acquiring an infection when a contact occurs with an infectious case, as well as the probability of developing symptoms (both mild and severe) for people who are vaccinated and become infected.

The implementation and parametrization of vaccines has changed over time as new information has become available. Originally, for the delta variant analyses associated with the roadmap [9] and original outbreak analyses, vaccines were implemented with single efficacy values for protection against infection, symptoms and severe disease that did not change over time, with the exception of a time-lag for immunity to develop (Table S5, Figure S4).

The vaccine's prevention of infection is approximated as "leaky", meaning that each person vaccinated has reduced but non-zero risk of becoming infected based on the vaccine efficacy (as opposed to an "all or nothing" vaccine, where 80% efficacy means that 80% of people have perfect protection and 20% have no protection).

Multiple vaccine interventions were implemented in the model, with each vaccine intervention defined by vaccine type and time between doses (e.g. AstraZeneca 12-weeks). People who received their first vaccination were assumed to receive their second at the scheduled time, and vaccine immunity (protection against infection and disease) was modelled to increase over time. The time to reach the estimated peak efficacies reported in Table S5 was dependent on vaccine type and time between doses, and the immunity profile assumed for the Pfizer 3, 6 and 8-week and the AstraZeneca 12 and 6-week vaccinations are shown in Figure S4.

Table S5: Original vaccine efficacy parameters against the delta variant, based on estimates for vaccines against the delta variant from Imperial College London, London School of Hygiene and Tropical Medicine and Warwick University from June 2021 [10].

Vaccine impact	Infection	Onward transmission	Symptoms	Hospitalization	ICU	Death
Overall protection: Pfizer 1	47%	33%	47%	71%	71%	71%
Overall protection: Pfizer 2	80%	56%	85%	87%	89%	92%
Overall protection: AstraZeneca 1	43%	24%	43%	69%	69%	69%
Overall protection: AstraZeneca 2	62%	45%	71%	86%	88%	90%

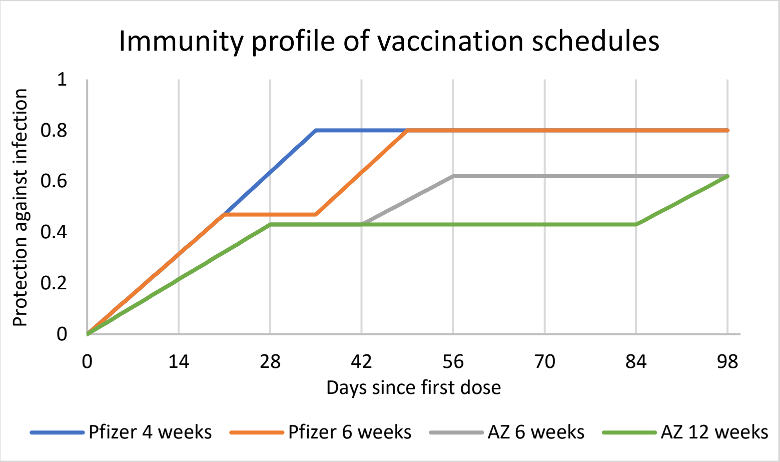

Figure S4: Vaccination immunity profile over time. Vaccinations were modelled according to vaccine type and time between doses and had a time-varying protection that depended on the vaccine type and time between doses.

However, but as more evidence has become available, the model implementation has since been updated and now includes waning of immunity over time. Currently, individuals are modelled to have a level of “neutralizing antibodies” (NAbs). NAbs can be acquired through either vaccination or infection, with different doses of different vaccines lead to different levels of NAbs. NAbs are then assumed to wane over time following an exponential function. A separate logistic relationship is then modelled that relate a person’s NAb levels to estimates for protection against infection, symptoms and severe disease [11]. For the Pfizer and AstraZeneca vaccines, the induced peak NAb levels following vaccination and rate of waning were calibrated to align with UK SAGE estimates [12] for protection against infection, symptoms and severe disease from the Delta variant (Figure S5, Table S6). The peak NAbs following a third dose were calibrated to produce the increase in protection from the published literature [13, 14] (Table S7).

Note that although cellular immune response is not modelled explicitly it is captured through the functions translating NAb levels into risk reduction against infection, symptomatic infection and severe disease; for example, even very low NAb levels result in protection against symptomatic and severe disease which is likely due to components of the immune response other than NAbs.

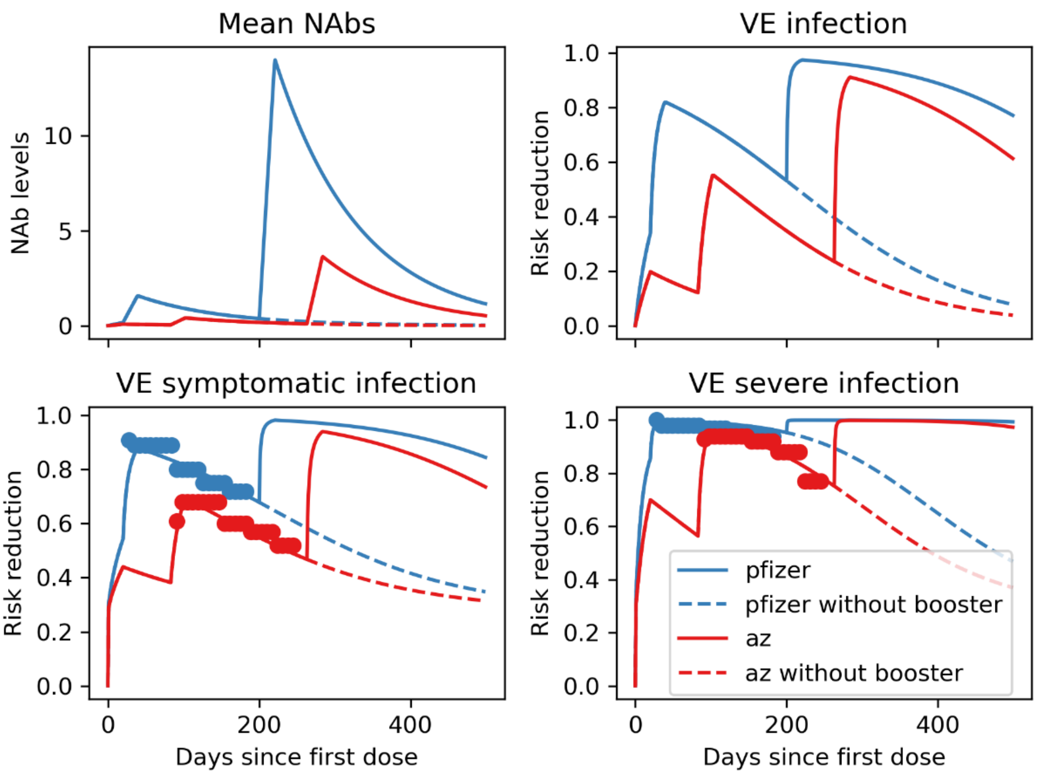

Figure S5: Estimated efficacy of Pfizer (blue) and AstraZeneca (red) vaccines against Delta variant over time. Time between consecutive Pfizer doses are assumed to be 3 weeks (second dose) and 5 months (third dose). Time between AstraZeneca vaccines is assumed to be 12 weeks, followed by a third dose of Pfizer 5 months later. curve assumes third dose of Pfizer. Dots correspond to estimated efficacy values from UK SAGE, Sep 2021 [12].

Table S6: Peak vaccine efficacy against Delta, based on implementation in **Figure S5**, and UK SAGE, Sep 2021 estimates [12].

	Peak protection against infection		Peak protection against symptomatic infection		Peak protection against severe disease	
Vaccine	*Dose 1*	*Dose 2*	*Dose 1*	*Dose 2*	*Dose 1*	*Dose 2*
Pfizer	35%	82%	55%	88%	86%	100%
AstraZeneca	21%	55%	45%	69%	71%	96%

Table S7: Relative effectiveness of Pfizer booster dose compared to effectiveness of two doses after five months, by vaccine [13, 14].

Vaccine received for first doses	Infection	Symptomatic infection	Severe infection
Pfizer	94%	94%	99.9%
AstraZeneca	88%	88%	99.7%

Virus strains

The model transmission parameters are calibrated to the variant in circulation at the time. The incubation period was shortened to a mean time from exposure to becoming infectious of 3.71 days, compared to 4.50 days for the wild type virus [15]. Disease prognoses (e.g., age-specific probability of requiring hospitalization, ICU or of dying) were updated to reflect the increased severity of the strain [16] (adjusted odds ratio for hospitalization, ICU and death of 2.08 relative to wild type; see appendix).

Model calibration

Model parameters for transmission and testing were calibrated to data on daily new detected cases, hospitalisations and ICU from the Delta COVID-19 epidemic wave in Melbourne over the July-September 2021 period [9]. The model was initialised with a population of 100,000 agents, and the overall transmission risk per contact (which multiplies the transmission probabilities in Table S1 for each layer), the per-day probability of a symptomatic individual seeking testing were varied such that the distribution of model outcomes for diagnoses, hospitalizations and number of tests was centred near the actual epidemic trajectory. For additional details see [9].

For this analysis, the model was initialized with only a single case in a school, as described in the main report, however the transmission and testing parameters were based on this previous calibration.

Disease prognosis

People in the model who became infected had an age-specific probability of becoming symptomatic or developing severe or critical disease. These probabilities are shown in Table S8 for unvaccinated people, and are modified according to vaccination status (Figure S5).

People who were infected and had severe or critical disease also had an age-specific probability of being in hospital or ICU, based on Knock et al. [17]. I.e. in the model, the probability of ICU given being in a critical condition is not necessarily 1, for example for people over 70 years.

Table S8: Age-specific susceptibility, disease progression and mortality risks for unvaccinated people, Delta variant.

Age bracket	Relative susceptibility*	Pr(symptomatic | infection)^	Pr(severe | infection)#	Pr(critical | infection)#	Pr(death | infection)#	Pr(hospital | severe)##	Pr(ICU | critical)##
0-4	0.34	0.533	0.016	0.00006	0.00002	1	1
5-9	0.34	0.533	0.016	0.00006	0.00002	1	1
10-14	0.34	0.533	0.016	0.00010	0.00002	1	1
15-19	1	0.533	0.016	0.00017	0.00002	1	1
20-24	1	0.679	0.045	0.002	0.00011	1	1
25-29	1	0.679	0.045	0.002	0.00011	1	1
30-34	1	0.679	0.076	0.004	0.00035	1	1
35-39	1	0.679	0.076	0.004	0.00035	1	1
40-44	1	0.679	0.109	0.009	0.00107	1	1
45-49	1	0.679	0.109	0.009	0.00107	1	1
50-54	1	0.679	0.158	0.030	0.00180	1	1
55-59	1	0.679	0.158	0.030	0.00180	1	1
60-64	1.24	0.803	0.308	0.050	0.00992	1	1

65-69	1.24	0.803	0.308	0.050	0.00992	1	1
70-74	1.47	0.803	0.440	0.090	0.05012	1	0.71
75-79	1.47	0.803	0.440	0.092	0.05012	1	0.71
80-89	1.47	0.803	0.550	0.297	0.10007	1	0.10
90+	1.47	0.803	0.550	0.297	0.12161	1	0.10

*Zhang et al. [18] found children <14 had 34% less susceptibility to adults, and people>65 years had 47% increased susceptibility
^Meta analysis; Sah et al. [19]
Victorian Department of Health Delta wave (2021) data
Knock et al. [17] used to calculate age-specific pr(hospitalization or ICU | infection with wild type); This implies that in the model, only a percentage of people with critical disease end up in ICU, according to age-specific pr(ICU|infection) from Knock et al.

Hospital length of stay

Length of stay in hospital was calculated separately for people who do or don't go to ICU. Victorian data was used from the Delta variant epidemic wave July-September 2021 (Figure S6). Data were separated by age but were not as granular as ICU data (Table S10 and Figure S7) due to small numbers (Vic only vs all of Australia data). For people in ICU, ICU days are included as hospital days.

Table S9: Values used to fit lognormal distribution number of ward days: Victorian data from Delta variant 2021 epidemic wave.

	Median	Percentile 05	Percentile 25	Percentile 75	Percentile 95
People who went to ICU					
0-59	3.2	0.0	1.4	9.0	18.0
60+	6.8	0.4	0.5	7.0	14.9
People who did not go to ICU					
0-59	4	1	1	8	13
60+	3	1	2	7	13

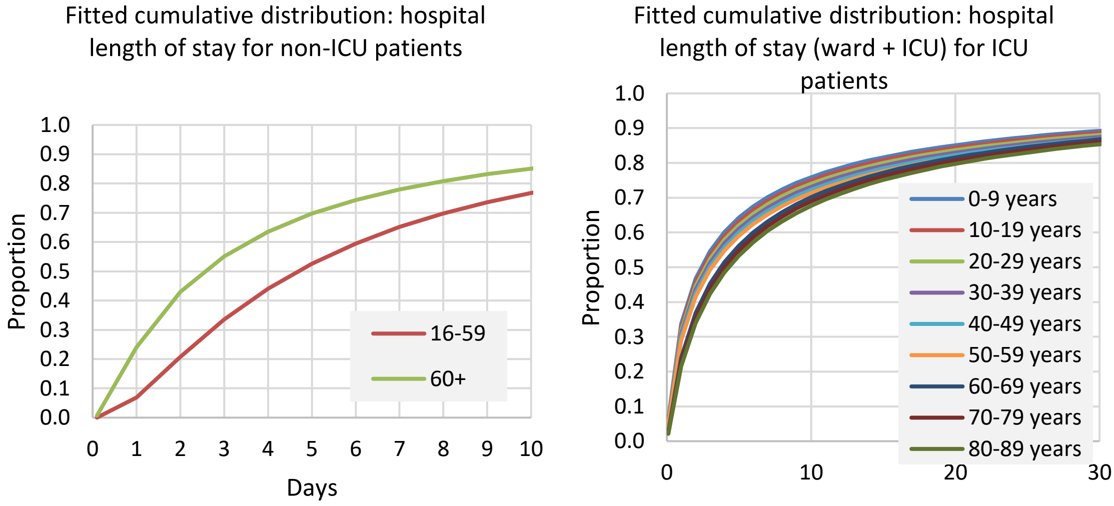

Figure S6: Distribution of length of stay in hospital by age, Victorian data from Delta variant 2021 epidemic wave. Left: people who did not go to ICU. Right: people who did go to ICU.

ICU length of stay

ICU length of stay was extracted from SPRINT SARI, in 10-year age categories, based on national data from the Delta variant epidemic waves July-September 2021. Linear regression was used to smooth median/IQR to ensure consistent pattern with increasing age (Table S10). Lognormal distributions were then fitted to the quantile estimates in Table S10, with the distributions (Figure S7) used as model inputs.

Table S10: Fitted values for ICU length of stay used in the model

	Fitted median	Fitted 25-percentile	Fitted 75-percentile
0-9 years	3.84	2.94	4.89
10-19 years	3.96	2.81	5.52
20-29 years	4.07	2.67	6.16
30-39 years	4.19	2.54	6.79
40-49 years	4.31	2.41	7.42
50-59 years	4.43	2.28	8.06
60-69 years	4.55	2.15	8.69
70-79 years	4.67	2.02	9.32
80-89 years	4.78	1.88	9.96

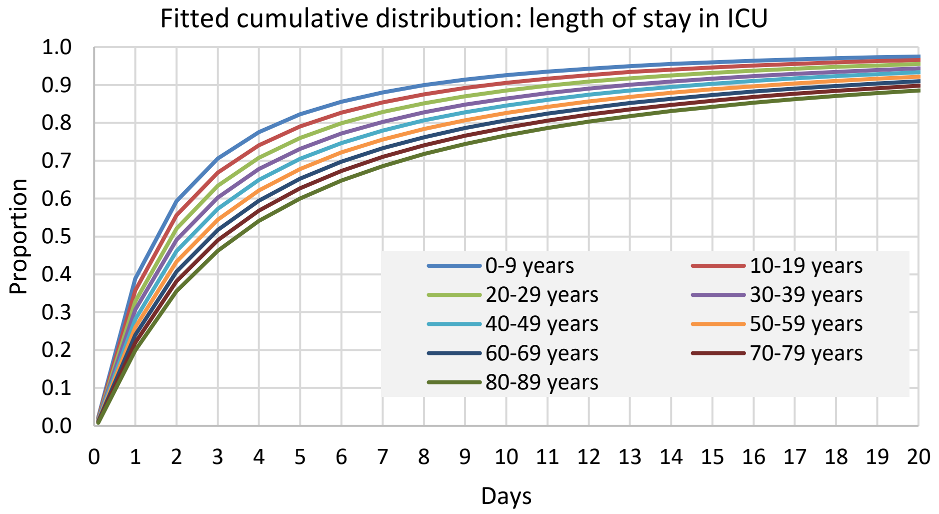

Figure S7: Distribution of length of stay in ICU by age, national data from Delta variant 2021 epidemic wave.

Policies

The policies being modelled apply to different contact networks in the model, and have been derived through calibration to past epidemic outbreaks in Victoria and NSW [6, 20, 21].

Table S11: Policy impacts in the model.

Policy area	Policy	Impact in model
Childcare	Authorized or vaccinated parents only	Reduce transmission by 60%. Based on 40% reduction in enrolment (authorized only component), 50% of remaining

		children are excluded with the vaccination policy, and an overall assumed additional 50% reduction due to NPIs ([0.6 + 0.4*0.5] *0.5).
Schools	Closed	Attendance reduced to 9% for vulnerable children
	Open with NPIs	Full attendance and 50% reduction in transmission risk in school setting. Applied independently per year level.
	Cohorting	When this policy is in place, the probability of transmission per school contact based on the proportion of time spent in person (e.g. 3/5 for three days per week). Applied to specific year levels.
Café/restaurant	Take-away only	30% of venues closed; venue capacity reduced to 0; 35% reduction in transmission risk per contact
	Outdoor only with 4sqm rule	80% reduction in number of contacts per person; 50% reduction in transmission risk per contact
	4 sqm rule	50% reduction in number of contacts per person
Pub/bar	Take-away only	30% of venues closed; venue capacity reduced to 0; 35% reduction in transmission risk per contact
	Outdoor only with 4sqm rule	80% reduction in number of contacts per person; 50% reduction in transmission risk per contact
	4 sqm rule	50% reduction in number of contacts per person
Retail	Essential only	20% of retail closed; 40% reduction in contacts for retail work that remains open
	4 sqm	50% reduction in contacts
Places of worship	Closed	Contacts in setting reduced to zero
	Outdoor only	80% reduction in transmission risk per contact
	4 sqm	30% reduction in transmission risk per contact
Community sport	Closed	Contacts set to zero
Outdoor gatherings	2 for exercise	50% reduction in transmission risk in social and public park networks
	<10 outdoors + no home visitors	20%, 40% and 40% relative reductions in transmission risk in community, social and public park networks
	<50 outdoors + 5 visitors at home	20% reduction in community and social contacts, 10% reduction in public park contacts
Non-retail work	Work from home if possible, construction closed	20% of workforce removed from work network; 40% relative reduction in transmission risk per work contact; 33% reduction in transmission risk per transport contact and community contact; 10% increase in household transmission risk
	Work from home if possible, construction open	13% of workforce removed from work network; 40% relative reduction in transmission risk per work contact; 20% reduction in transmission risk per transport contact and community contact; 10% increase in household transmission risk
Entertainment	Closed	Contacts turned off
	Outdoor only, 10 per group	70% reduction in number of contacts per person; 80% reduction in transmission risk per contact
	4 sqm	70% reduction in number of contacts per person
Mobility	5km	30-70% reduction on community transmission
	10km	20% and 50% reduction in community and transport transmission risks
Masks	Mandatory	30% reduction in transmission risk in work, entertainment; transport, aged care settings; 25% reduction in community, social, public parks and places of worship; 20% reduction in schools; 10% reduction in cafes, restaurants pubs and bars
	Indoors only	30% reduction in transmission risk in work, entertainment; transport, aged care settings; 25% reduction in places of worship; 20% reduction in schools; 10% reduction in cafes, restaurants pubs and bars

Model implementation

The model is implemented in Python and is available in an open access repository (https://github.com/InstituteforDiseaseModeling/covasim), where any elements can be adapted to a particular context or research question. Individual simulations can be run on a personal computer without requiring other software, with a single simulation taking between 5-20 seconds per 30-day period, depending on the epidemic size (with bigger outbreaks taking longer to simulate as more contact tracing, quarantine or isolation algorithms need to be applied). Therefore, while testing can be conducted locally, to run 1000 simulations for each scenario requires parallel runs on a server with a larger number of processors.

Parameters used to define scenarios

Scenario type 1: prospective outbreak analyses (elimination strategy context)

These scenarios were applied in the context of no community cases, to assess the outbreak risk associated with incursions into the community from hotel quarantine or other sources. This type of scenario was useful for policy in the context of an elimination strategy.

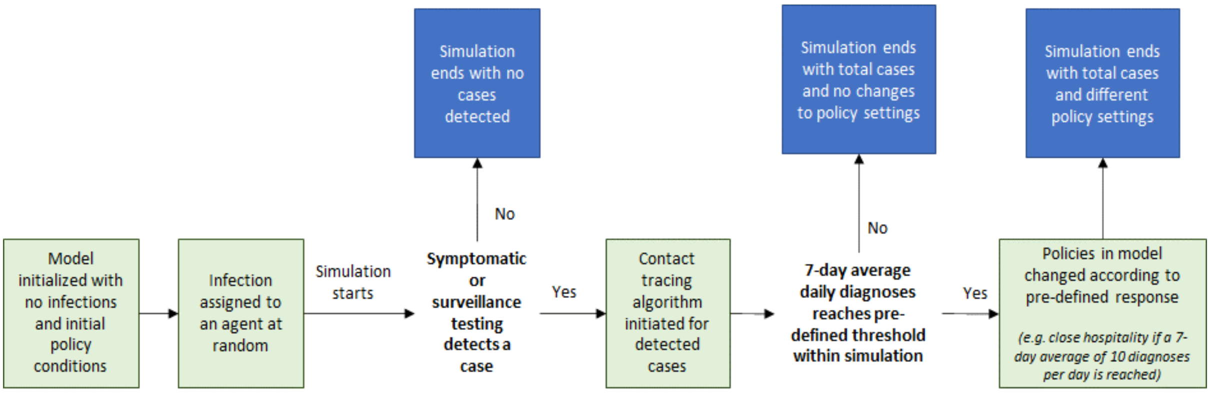

Figure S8: Schematic of outbreak analysis with dynamic policy changes.

Parameters that were varied to define scenarios were:

- Initial policy conditions: any combination of policies from Table S11.
- Number of agents infected at t=0 to start outbreak: typically this would be one, but in specific cases where it was known that multiple incursions had occurred simultaneously, the model could be initialized with multiple simultaneous incursions.
- Contact tracing algorithm on detection of cases: defined by values for the parameters in Table S3, setting the probability of detecting contacts through different networks and the likelihood of people complying with quarantine or isolation instructions.
- Thresholds for triggering policy changes: pairs of inputs for *(7-day average daily detected cases, policies)*. In the model, if the 7-day average daily detected cases were reached, it would update the set of policies. The new policies could be any collection of those in Table S11. Multiple thresholds could also be used, for example allowing combinations such as "if the 7-day average reaches 10 then close hospitality, if it reaches 20 additionally close schools and non-retail work".

The principal output measure for each scenario, defined by the inputs above, was the percentage of simulations where the epidemic reached different sizes over a fixed period (e.g., 90 days).

Scenario type 2: reactive outbreak analyses (elimination strategy context)

These scenarios were similar to scenario type 1 but were applied during specific outbreaks, rather than when no outbreaks were occurring. They were implemented similarly to scenario type 1 but included additional constraints on parameters to ensure the simulated outbreak accurately represented the specific real world outbreak:

- Initial policy conditions: those in place when outbreak commenced.
- Number of agents infected at t=0 to start outbreak: based on number of incursions that occurred in the real world.
- Contact tracing algorithm on detection of cases: extracted from government contact tracing databases.
- Thresholds for triggering policy changes: for the past, policies set to change over time as occurred in the real world. For the future, additional trigger thresholds and/or policy changes could be implemented as scenarios.

Scenario options were therefore restricted to future policy changes. The principal output measure for each scenario, defined by the inputs above, was the percentage of simulations where the epidemic reached different sizes over a fixed period (e.g., 90 days). The difference between this and scenario type 1 was that simulations were excluded from these calculations if they did not align with the past observed detected cases (e.g. if 20 cases were detected in the first week in the real world, then any simulated outbreak not meeting that condition to some tolerance was discarded).

Scenario type 3: easing restrictions (elimination strategy context)

These scenarios were useful at the end of an outbreak or epidemic wave, when scenarios around easing restrictions and resurgence risk were being explored. They were implemented analogously to scenario type 2, but over a much longer time scale. This involved simulating outbreaks that became large, and in the model implementing various policy and behavioural changes at different time points according to data on what occurred. Specifically:

- Initial policy conditions: those in place when outbreak commenced.
- Number of agents infected at t=0 to start outbreak: could be varied depending on the nature of the outbreak (e.g. if frequent cases were entering the community from interstate this could be a larger number).
- Contact tracing algorithm on detection of cases: extracted from government contact tracing databases; *able to decrease in efficacy with epidemic scale*.
- Thresholds for triggering policy changes: for the past, policies set to change over time in the model as occurred in the real world.

The model was calibrated analogously to the scenario type 2, where simulations were only retained if they were consistent with the actual outbreak. This resulted in a collection of simulations that met the conditions and past policy changes observed in the real world, which could then be used for further scenario analyses.

Of the retained simulations, the main outcome measure for each scenario was the probability of reaching >N diagnoses per day following the easing of restrictions (i.e., "resurgence risk"), and how this varied according to the timing and extent that restrictions were eased.

Scenario type 4: health system utilization (control strategy context)

These scenarios were useful when a control strategy rather than elimination strategy was in place. Scenarios were implemented analogously to scenario type 3, however with different key outputs being considered (e.g. hospital and ICU demand rather than the number of cases).

Supplement references

1. Australian Bureau of Statistics (ABS): **Victorian population data https://www.abs.gov.au/statistics/people/population/national-state-and-territory-population/latest-release**. March 2021.
2. Australian Bureau of Statistics (ABS): **Australian Bureau of Statistics 2016, Census of Population and Housing, TableBuilder. Findings based on use of ABS TableBuilder data. https://www.abs.gov.au/ausstats/abs@.nsf/web+pages/Citing+ABS+Sources#TableBuilder i**. 2020.
3. Australian Bureau of Statistics (ABS): **Education statistics. https://www.abs.gov.au/statistics/people/education/schools/latest-release#data-download** 2020.
4. Victorian Government: **Government schools average class size. https://discover.data.vic.gov.au/dataset/government-schools-average-class-size-primary-classes-feb-victoria**. 2021.
5. OECD: **Education at a Glance 2021**; 2021.
6. Scott N, Palmer A, Delport D, Abeysuriya R, Stuart R, Kerr CC, Mistry D, Klein DJ, Sacks-Davis R, Heath K *et al*: **Modelling the impact of relaxing COVID-19 control measures during a period of low viral transmission**. *Medical Journal of Australia* 2021, **214**(2):79-83.
7. Arevalo-Rodriguez I, Buitrago-Garcia D, Simancas-Racines D, Zambrano-Achig P, Del Campo R, Ciapponi A, Sued O, Martinez-Garcia L, Rutjes AW, Low N: **False-negative results of initial RT-PCR assays for COVID-19: a systematic review**. *PloS one* 2020, **15**(12):e0242958.
8. Muhi S, Tayler N, Hoang T, Ballard SA, Graham M, Rojek A, Kwong JC, Trubiano JA, Smibert O, Drewett G: **Multi-site assessment of rapid, point-of-care antigen testing for the diagnosis of SARS-CoV-2 infection in a low-prevalence setting: A validation and implementation study**. *The Lancet Regional Health-Western Pacific* 2021, **9**:100115.
9. Abeysuriya R, Delport D, Sacks-Davis R, Hellard M, Scott N: **Modelling the Victorian roadmap. 18 September 2021.** *[Burnet Institute Public Health Report]*.
10. UK Government Coronavirus Information: **SPI-M-O: Summary of further modelling of easing restrictions – Roadmap Step 4. Available from: https://assets.publishing.service.gov.uk/government/uploads/system/uploads/attachment_data/file/1075056/S1287_SPI-M-O_Summary_Roadmap_step_4.pdf**. 9th June 2021.
11. Cohen JA, Stuart RM, Rosenfeld K, Lyons H, White M, Kerr CC, Klein DJ, Famulare M: **Quantifying the role of naturally- and vaccine-derived neutralizing antibodies as a correlate of protection against COVID-19 variants**. *medRxiv* 2021:2021.2005.2031.21258018.
12. UK SAGE: **Public Health England estimates for duration of protection of covid-19 vaccines against clinical disease. Accessed 1 Feb 2022 from https://assets.publishing.service.gov.uk/government/uploads/system/uploads/attachment_data/file/1017309/S1362_PHE_duration_of_protection_of_COVID-19_vaccines_against_clinical_disease.pdf**. 9 Sep 2021.
13. Bar-On YM, Goldberg Y, Mandel M, Bodenheimer O, Freedman L, Kalkstein N, Mizrahi B, Alroy-Preis S, Ash N, Milo R: **Protection of BNT162b2 vaccine booster against Covid-19 in Israel**. *New England Journal of Medicine* 2021, **385**(15):1393-1400.
14. Barda N, Dagan N, Cohen C, Hernán MA, Lipsitch M, Kohane IS, Reis BY, Balicer RD: **Effectiveness of a third dose of the BNT162b2 mRNA COVID-19 vaccine for preventing severe outcomes in Israel: an observational study**. *The Lancet* 2021, **398**(10316):2093-2100.
15. Li B, Deng A, Li K, Hu Y, Li Z, Xiong Q, Liu Z, Guo Q, Zou L, Zhang H: **Viral infection and transmission in a large well-traced outbreak caused by the Delta SARS-CoV-2 variant**. *Nature Communications* 2022, **13**(1):460.
16. Fisman D, Tuite A: **Evaluation of the relative virulence of novel SARS-CoV-2 variants: a retrospective cohort study in Ontario, Canada**. *Canadian Medical Association Journal* 2021*,* **193**(42):E1619-1625.
17. Knock ES, Whittles LK, Lees JA, Perez-Guzman PN, Verity R, FitzJohn RG, Gaythorpe KA, Imai N, Hinsley W, Okell LC: **Key epidemiological drivers and impact of interventions in the 2020 SARS-CoV-2 epidemic in England**. *Science Translational Medicine* 2021, **13**(602):eabg4262.
18. Zhang J, Litvinova M, Liang Y, Wang Y, Wang W, Zhao S, Wu Q, Merler S, Viboud C, Vespignani A: **Changes in contact patterns shape the dynamics of the COVID-19 outbreak in China**. *Science* 2020, **368**(6498):1481-1486.

19. Sah P, Fitzpatrick MC, Zimmer CF, Abdollahi E, Juden-Kelly L, Moghadas SM, Singer BH, Galvani AP: **Asymptomatic SARS-CoV-2 infection: A systematic review and meta-analysis**. *Proceedings of the National Academy of Sciences* 2021, **118**(34).
20. Abeysuriya RG, Delport D, Stuart RM, Sacks-Davis R, Kerr CC, Mistry D, Klein DJ, Hellard M, Scott N: **Preventing a cluster from becoming a new wave in settings with zero community COVID-19 cases**. *BMC Infectious Diseases* 2022, 22(1):1-15.
21. Abeysuriya R, Delport D, Hellard M, Scott N: **Estimating risks associated with early reopening in Victoria. 26 September 2020.** *[Burnet Institute Public Health Report]*.
